# Chemotherapy-related hyperbilirubinemia in pediatric acute lymphoblastic leukemia: a genome-wide association study from the AIEOP-BFM ALL study group

**DOI:** 10.1186/s13046-022-02585-x

**Published:** 2023-01-13

**Authors:** Stefanie V. Junk, Elke Schaeffeler, Martin Zimmermann, Anja Möricke, Rita Beier, Peter Schütte, Birthe Fedders, Julia Alten, Laura Hinze, Norman Klein, Andreas Kulozik, Martina U. Muckenthaler, Rolf Koehler, Arndt Borkhardt, Jayaram Vijayakrishnan, David Ellinghaus, Michael Forster, Andre Franke, Astrid Wintering, Christian P. Kratz, Martin Schrappe, Matthias Schwab, Richard S. Houlston, Gunnar Cario, Martin Stanulla

**Affiliations:** 1 Department of Pediatric Hematology and Oncology, Hannover Medical School, Carl-Neuberg-Str. 1, 30625 Hannover, Germany; 2 Margarete-Fischer-Bosch Institute of Clinical Pharmacology, Stuttgart, Germany; 3 Department of Pediatrics, University Hospital Schleswig-Holstein, Kiel, Germany; 4 Department of Pediatric Hematology, Oncology and Immunology, University of Heidelberg, Heidelberg, Germany; 5 Department of Human Genetics, University of Heidelberg, Heidelberg, Germany; 6 Clinic for Pediatric Oncology, Hematology, and Clinical Immunology, Medical Faculty, Heinrich-Heine-University, Düsseldorf, Germany; 7 Division of Genetics and Epidemiology, The Institute of Cancer Research, Sutton, Surrey, UK; 8 Institute of Clinical Molecular Biology, Kiel University, Kiel, Germany; 9 Departments of Clinical Pharmacology, and of Biochemistry and Pharmacy, University of Tuebingen, Tuebingen, Germany; 10 Cluster of Excellence iFIT (EXC 2180) “Image-Guided and Functionally Instructed Tumor Therapies”, University of Tuebingen, Tuebingen, Germany; 11 German Cancer Consortium (DKTK) and German Cancer Research Center (DKFZ) Partner Site Tübingen, Tübingen, Germany

**Keywords:** Childhood acute lymphoblastic leukemia, Treatment-related toxicity, Hepatotoxicity, Hyperbilirubinemia, *UGT1A*

## Abstract

**Background:**

Characterization of clinical phenotypes in context with tumor and host genomic information can aid in the development of more effective and less toxic risk-adapted and targeted treatment strategies. To analyze the impact of therapy-related hyperbilirubinemia on treatment outcome and to identify contributing genetic risk factors of this well-recognized adverse effect we evaluated serum bilirubin levels in 1547 pediatric patients with acute lymphoblastic leukemia (ALL) and conducted a genome-wide association study (GWAS).

**Patients and methods:**

Patients were treated in multicenter trial AIEOP-BFM ALL 2000 for pediatric ALL. Bilirubin toxicity was graded 0 to 4 according to the Common Toxicity Criteria (CTC) of the National Cancer Institute. In the GWAS discovery cohort, including 650 of the 1547 individuals, genotype frequencies of 745,895 single nucleotide variants were compared between 435 patients with hyperbilirubinemia (CTC grades 1-4) during induction/consolidation treatment and 215 patients without it (grade 0). Replication analyses included 224 patients from the same trial.

**Results:**

Compared to patients with no (grade 0) or moderate hyperbilirubinemia (grades 1-2) during induction/consolidation, patients with grades 3-4 had a poorer 5-year event free survival (76.6 ± 3% versus 87.7 ± 1% for grades 1-2, *P* = 0.003; 85.2 ± 2% for grade 0, *P* < 0.001) and a higher cumulative incidence of relapse (15.6 ± 3% versus 9.0 ± 1% for grades 1-2, *P* = 0.08; 11.1 ± 1% for grade 0, *P* = 0.007). GWAS identified a strong association of the rs6744284 variant T allele in the *UGT1A* gene cluster with risk of hyperbilirubinemia (allelic odds ratio (OR) = 2.1, *P* = 7 × 10^− 8^). TT-homozygotes had a 6.5-fold increased risk of hyperbilirubinemia (grades 1-4; 95% confidence interval (CI) = 2.9-14.6, *P* = 7 × 10^− 6^) and a 16.4-fold higher risk of grade 3-4 hyperbilirubinemia (95% CI 6.1-43.8, *P* = 2 × 10^− 8^). Replication analyses confirmed these associations with joint analysis yielding genome-wide significance (allelic OR = 2.1, *P* = 6 × 10^− 11^; 95% CI 1.7-2.7). Moreover, rs6744284 genotypes were strongly linked to the Gilbert’s syndrome-associated UGT1A1*28/*37 allele (r^2^ = 0.70), providing functional support for study findings. Of clinical importance, the rs6744284 TT genotype counterbalanced the adverse prognostic impact of high hyperbilirubinemia on therapy outcome.

**Conclusions:**

Chemotherapy-related hyperbilirubinemia is a prognostic factor for treatment outcome in pediatric ALL and genetic variation in *UGT1A* aids in predicting the clinical impact of hyperbilirubinemia.

**Trial registration:**

http://www.clinicaltrials.gov; #NCT00430118.

**Supplementary Information:**

The online version contains supplementary material available at 10.1186/s13046-022-02585-x.

## Background

The systematic evaluation of tumor and host genomic information can help to identify new predictive markers and cancer vulnerabilities and lead to novel effective and less toxic risk-adapted and targeted treatment strategies [1]. During the last five decades, treatment of pediatric patients with acute lymphoblastic leukemia (ALL) has significantly improved and is one of the success stories in clinical oncology [2]. However, although most children diagnosed with ALL can be cured by contemporary clinical protocols [2, 3], benefits in survival come with acute and long-term adverse effects. This may complicate administration of therapy or impact on health and quality of life during and after completion of treatment [2, 3]. Thus, there is continuing medical need to improve risk assessment and tailored treatment for children and adolescents with ALL.

Hepatotoxicity, mainly captured in clinical trials by assessment of hyperbilirubinemia and transaminasemia, is a well-known complication frequently occurring in the early phases of ALL treatment [4, 5]. Surprisingly, detailed reports describing incidence and impact of hepatotoxicity on the overall outcome of pediatric ALL are lacking. A recent study including 1872 pediatric ALL patients treated according to the ALL IC-BFM 2002 protocol reported 934 grade 3 or above events of hepatotoxicity according to the National Cancer Institute (NCI) Common Toxicity Criteria (CTC; for details see Suppl. Methods section) [6] in 527 individuals (28%) during the entire therapy [5]. A previous single institution study reported that event-free survival (EFS) was rarely influenced by hyperbilirubinemia, whereas treatment modifications including delays were common [4]. Overall, 17% of patients in the latter study had bilirubin levels equal or above CTC grade 3 at least once in the course of therapy [4]. Another study determined such high levels in 10, 2 and 15% of patients during induction, consolidation and maintenance phases, respectively [7].

High total serum bilirubin levels – with or without transaminasemia – may indicate liver dysfunction upon exposure to various antileukemic drugs including L-asparaginase or antimetabolites [8–10]. However, in this context the biological basis of hyperbilirubinemia is poorly understood. In the last decade, genome-wide association studies (GWAS) enabled a refined risk assessment of many diseases – including pediatric ALL – by identifying risk-associated genetic variants [11–13].

In the present study, we evaluated the clinical impact of hyperbilirubinemia in a large cohort of pediatric ALL patients and applied a GWAS approach to identify genetic variants influencing chemotherapy-related hyperbilirubinemia.

## Methods

### Study individuals

Patients included were 1 to 18 years of age at diagnosis of ALL and enrolled in the German part of the European AIEOP-BFM ALL 2000 multicenter clinical trial for frontline treatment of pediatric ALL from August 1999 to November 2005 [14–16] (for treatment details see Suppl. Table 1). Primary patient selection criteria were the availability of bilirubin toxicity gradings for induction or consolidation – protocols IA/IB – and, for the assembly of our GWAS discovery cohort, the availability of genome-wide germline genotyping information (Suppl. Fig. 1) obtained during a previous project [13]. When available, we also assessed bilirubin gradings related to subsequent treatment phases (extracompartment therapy, high-risk block treatment, and re-intensification protocols II and III). No information was available for maintenance treatment. For further details including statistical analyses, see Suppl. Methods section.

### Toxicity definitions

As part of the routine safety management, toxicity was assessed for all treatment elements except for interim maintenance and maintenance phases. Considering 17.1 μmol/L as the upper normal limit (UNL), total bilirubin serum levels were graded according to the CTC of the NCI, version 2 [6] (for details see Suppl. Methods section).

### Genotyping

In the discovery cohort, DNA obtained from bone marrow samples in morphological remission was genotyped on Human Omni1-Quad v1 arrays (Illumina, San Diego, CA, USA) as previously described [13]. Genotype information on rs6744284 for replication purposes was derived from a preceding analysis using Affymetrix Genome-wide Human SNP arrays 5.0 (Affymetrix, South San Francisco, CA, USA) [17].

Out of the discovery cohort, we genotyped 544 patients with additional DNA available for UGT1A1*28/*37 variations using a pre-developed KASP assay (LGC Biosearch Technologies, Hoddesdon, UK).

### Genome-wide association study

GWAS conduction, including data pruning, association testing with plink 1.9 (www.cog-genomics.org/plink/1.9/), evaluation and plotting, was realized with an in-house developed R (3.6.0) script using RStudio (1.0.143). A strict quality control was performed prior to association testing. For details on data pruning, association testing, genotype imputation and further statistical analyses see Suppl. Methods section.

## Results

### Clinical characterization of hyperbilirubinemia

Within the 1547 patients of the AIEOP ALL-BFM 2000 study population with available toxicity information, 540 (34.9%) had normal and 1007 (65.1%) had increased bilirubin levels during induction/consolidation (protocols IA/IB) (Table 1). This included 825 (53.3%) patients with moderate hyperbilirubinemia (grades 1-2; 707 during induction, 575 during consolidation, 355 in both phases) and 182 (11.8%) with high hyperbilirubinemia (grades 3-4; 158 reports during induction, 50 during consolidation, 26 in both phases). Comparing patients without to patients with hyperbilirubinemia (grade 0 vs. grades 1-4), we noticed a larger proportion of older patients (*P* < 0.001) and more T cell ALL patients (*P* = 0.022) among those affected. The group of B cell ALL patients exhibiting hyperbilirubinemia contained fewer hyperdiploid patients (*P* = 0.002). However, no differences with regard to other genetic subgroups were observed (*ETV6-RUNX1, BCR-ABL1* and *KMT2A-AFF1*) (Table 1). Considering the entire course of therapy, 245 patients (16.0%) had high hyperbilirubinemia in at least one treatment element. We determined a median time to protocol day 78 (after completion of induction and consolidation) of 89 ± 11 days (range 64-185 days), analyzing 1453 of 1547 individuals of our study population with available information. Compared to patients with moderate or no hyperbilirubinemia, patients with hyperbilirubinemia grades 3-4 experienced more therapy delays, requiring 91 days (range 64-174 days) to complete induction/consolidation vs. 88 days (range 70-154 days) for grades 1-2 and 89 days (range 70-185 days) for grade 0 (*P* = 0.002). No alterations of therapy in response to hyperbilirubinemia were noted.

**Table 1:** Characteristics of 1547 patients according to serum bilirubin levels during induction/consolidation therapy for acute lymphoblastic leukemia

		Patients without hyperbilirubinemia		Patients with hyperbilirubinemia		***P***^**a**^
		(*n* = 540)	(%)	(*n* = 1007)	(%)	
Sex	Male	300	(56%)	561	(56%)	
	Female	240	(44%)	446	(44%)	0.954
Age at diagnosis of ALL [y]	< 6	372	(69%)	483	(48%)	
	≥6 < 10	93	(17%)	205	(20%)	
	≥10	75	(14%)	319	(32%)	< 0.001
Immunophenotype	B cell ALL	461	(85%)	833	(83%)	
	T cell ALL	55	(10%)	146	(14%)	0.022
	Other/not characterized^b^	24	(4%)	28	(3%)	
White blood cell count at diagnosis of ALL [/μL]	< 10,000	261	(48%)	488	(48%)	
	≥10,000 < 50,000	187	(35%)	323	(32%)	
	≥50,000 < 100,000	52	(10%)	95	(9%)	
	≥100,000	40	(7%)	100	(10%)	0.364
	Unknown	0	(0%)	1	(0%)	
CNS positivity^c^	No	508	(94%)	932	(93%)	
	Yes	13	(2%)	33	(3%)	0.326
	Unknown	19	(4%)	42	(4%)	
Hyperdiploidy^d^	No	303	(56%)	627	(62%)	
	Yes	105	(19%)	138	(14%)	0.002
	Unknown	132	(24%)	242	(24%)	
*ETV6-RUNX1* rearrangement	Negative	380	(70%)	688	(68%)	
	Positive	120	(22%)	231	(23%)	0.636
	Unknown	40	(7%)	88	(9%)	
*BCR-ABL1* rearrangement	Positive	8	(1%)	20	(2%)	
	Negative	500	(93%)	941	(93%)	0.500
	Unknown	32	(6%)	46	(5%)	
*KMT2A-AFF1* rearrangement	Positive	2	(0%)	4	(0%)	
	Negative	471	(87%)	890	(88%)	0.948
	Unknown	67	(12%)	113	(11%)	
Prednisone response^e^	Good	492	(91%)	898	(89%)	
	Poor	40	(7%)	96	(10%)	0.162
	Unknown	8	(1%)	13	(1%)	
MRD risk group^f^	Standard	223	(41%)	428	(43%)	
	Intermediate	251	(46%)	432	(43%)	
	High	32	(6%)	72	(7%)	0.393
	Unknown	34	(6%)	75	(7%)	
Final risk group^g^	Standard	167	(31%)	318	(32%)	
	Intermediate	303	(56%)	532	(53%)	
	High	69	(13%)	156	(15%)	0.282
	Other/Unknown	1	(0%)	1	(0%)	
Maximum transaminase levels during protocols IA/IB^h^	CTC grade 0	71	(13%)	25	(2%)	
	CTC grades 1-2	249	(46%)	429	(43%)	
	CTC grades 3-4	212	(39%)	553	(55%)	< 0.001
	Unknown	8	(1%)	0	(0%)	
Maximum bilirubin levels during protocol IA^i^	CTC grade 0	499	(92%)	121	(12%)	
	CTC grades 1-2	0	(0%)	707	(70%)	
	CTC grades 3-4	0	(0%)	158	(16%)	< 0.001
	Unknown	41	(8%)	21	(2%)	
Maximum bilirubin levels during protocol IB^i^	CTC grade 0	501	(93%)	342	(34%)	
	CTC grades 1-2	0	(0%)	575	(57%)	
	CTC grades 3-4	0	(0%)	50	(5%)	< 0.001
	Unknown	39	(7%)	40	(4%)	
Maximum bilirubin levels during protocol IA/IB^i^	CTC grade 0	540	(100%)	0	(0%)	
	CTC grades 1-2	0	(0%)	825	(82%)	
	CTC grades 3-4	0	(0%)	182	(18%)	< 0.001
Maximum bilirubin levels during the entire course of therapy^j^	CTC grade 0	412	(76%)	0	(0%)	
	CTC grades 1-2	123	(23%)	767	(76%)	
	CTC grades 3-4	5	(1%)	240	(24%)	< 0.001

*Abbreviations*: *CNS* central nervous system, *CTC* Common Toxicity Criteria of the National Cancer Institute version 2, *UNL* Upper normal limit

^a^*P*-values resulting from *X*^2^ or Fisher’s exact test: Patients of the study cohort with hyperbilirubinemia, i.e. bilirubin levels > 17.1 μmol/L (UNL) during induction and/or consolidation (protocols IA/IB) of the AIEOP-BFM ALL protocol (CTC grades 1-4, cases) versus patients with normal levels ≤17.1 μmol/L (CTC grade 0, controls)

^b^ One patient was diagnosed with acute undifferentiated leukemia and no immunophenotype information was available for 51 patients

^c^ CNS negative, puncture nontraumatic without leukemic blasts in the cerebrospinal fluid (CSF) after cytocentrifugation; CNS positive, puncture nontraumatic with > 5 leukocytes /μL in the CSF with identifiable blasts

^d^ Defined by cytogenetics (> 50 chromosomes) or by flow cytometric analyses of the ratio of DNA content of leukemic G0/G1 cells to normal diploid lymphocytes (≥1.16)

^e^ Good < 1000 leukemic blasts/μL peripheral blood on treatment day 8; poor ≥1000 blasts/μL

^f^ Risk stratification based on minimal residual disease (MRD) analysis for ERG: Standard risk, MRD-negative on treatment day 33 and 78; high risk, leukemic cell load ≥5 × 10^−4^ on treatment day 78; all other results correspond to intermediate risk

^g^ Treatment group according to risk stratification including all relevant diagnostic parameters

^h^ Toxicity grading of the alanine and aspartate transaminase serum activity levels during induction/consolidation (protocols IA/IB) was according to CTC, considering 20 U/L as the UNL

^i^ Bilirubin toxicity grading during induction/consolidation (protocols IA/IB) was according to the CTC, with grade 0 corresponding to total serum levels ≤UNL, grade 1 to levels >UNL to 1.5xUNL, grade 2 levels > 1.5x UNL to 3.0x UNL, grade 3 levels > 3.0x UNL to 10.0x UNL and grade 4 to levels > 10.0x UNL

^j^ The highest individual bilirubin toxicity level throughout the entire treatment course under investigation (compare Suppl. Fig. 4). Toxicity grading was as above (CTC)

**Fig. 1:**
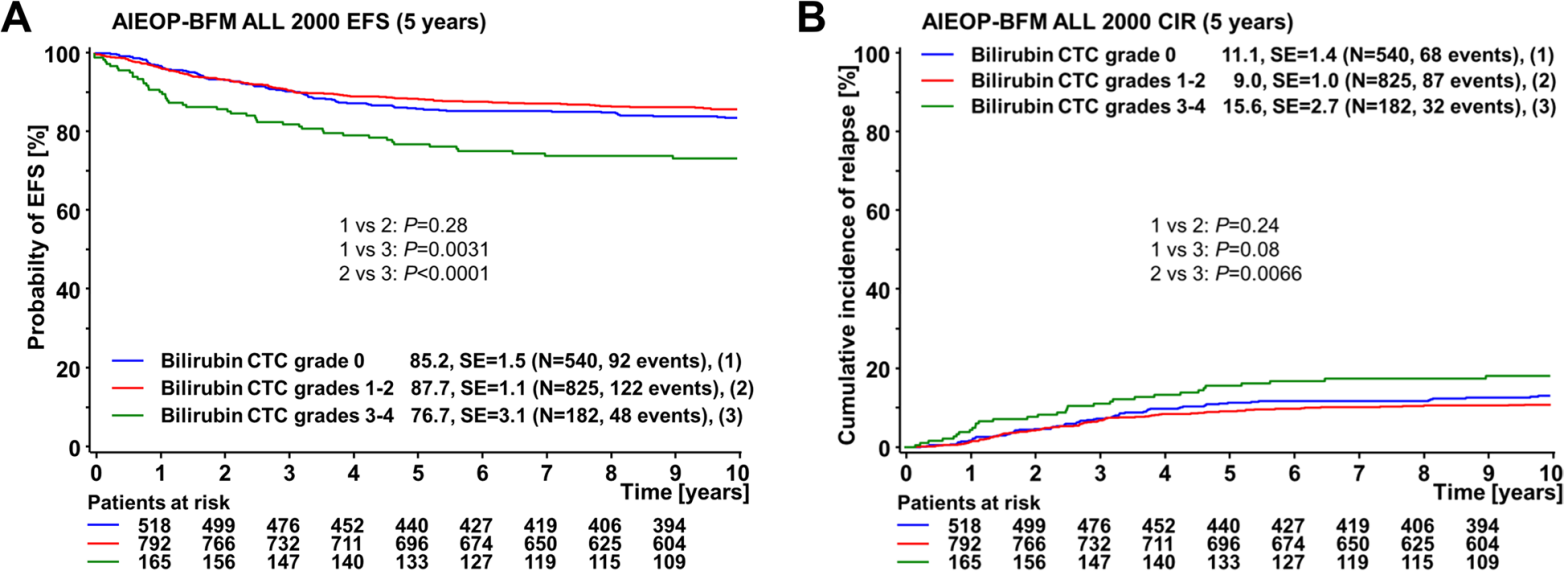
**A** Probability of 5 year event free survival (EFS) [%] and (**B**) corresponding cumulative incidence of relapse (CIR) according to the maximum total bilirubin toxicity during induction/consolidation – protocols IA/IB –in the in the AIEOP ALL-BFM 2000 study population with available bilirubin information (*n* = 1547 patients). Bilirubin toxicity grading was according to the Common Toxicity Criteria (CTC) of the National Cancer Institute, version 2; standard error (SE) and the number of included individuals are indicated for each category

In outcome analyses, patients with high hyperbilirubinemia (grades 3-4) during induction/consolidation fared significantly worse compared to patients with moderate or no hyperbilirubinemia: 5-year EFS 76.7 ± 3% vs. 87.7 ± 1% (*P* < 0.0001), and vs. 85.2 ± 2% (*P* = 0.0031), respectively (Fig. 1A). The corresponding 5-year cumulative incidences of relapse (CIR) were 15.6 ± 3% for high, 9.0 ± 1% for moderate hyperbilirubinemia, and 11.1 ± 1% for patients without hyperbilirubinemia (Fig. 1B).

In our study cohort, 1443 (93%) patients were observed with elevated hepatic transaminase activity levels (grades 1-4) during induction/consolidation (Table 1), 765 of which demonstrated grades 3-4. Transaminase levels were positively correlated with hyperbilirubinemia and, in particular, patients with high hyperbilirubinemia were at risk for concurrent high transaminase levels (grades 3-4) compared to the remaining patients (70% vs. 40%, odds ratio (OR) = 21.3, 95% confidence interval (CI) = 5.1-88.2, *P* = 2.53 × 10^− 5^) (Suppl. Table 2).

Interestingly, 5-year EFS and CIR did not differ between patients with moderate (grades 1-2), high (grades 3-4) or absent transaminasemia during induction/consolidation (Suppl. Fig. 2A and B). The 5-year EFS of patients with concurrent high hyperbilirubinemia and high transaminasemia was 76.2 ± 4% and 78.1 ± 6% in patients with high hyperbilirubinemia accompanied by moderate or no transaminasemia (grades 0-2, *P* = 0.63; Suppl. Fig. 2C). The corresponding CIR were 17.5 ± 3% and 11.0 ± 4% (*P* = 0.13; Suppl. Fig. 2D).

Multivariate analyses including established prognostic factors in AIEOP-BFM trials identified high hyperbilirubinemia as an independent predictor of outcome, while severe transaminasemia (CTC grade 4) did not demonstrate an impact here (Table 2).

**Table 2:** Estimated hazard ratios^a^ from the multivariable Cox proportional model on event-free survival and hazard of relapse in patients of the study cohort

Variable	Event		Relapse	
	Hazard Ratio (95% CI^**a**^)	***P(X***^***2***^***)***	Hazard Ratio (95% CI^**a**^)	***P(X***^***2***^***)***
Bilirubin CTC grades 3-4^b^	1.67 (1.21-2.30)	0.002	1.49 (1.00-2.21)	0.049
ALT/AST CTC grade 4^c^	0.88 (0.56-1.38)	0.575	1.02 (0.61-1.69)	0.955
MRD standard risk^d^	0.49 (0.34-0.69)	< 0.001	0.51 (0.34-0.77)	0.001
MRD high risk^d^	4.07 (2.87-5.78)	< 0.001	4.17 (2.72-6.38)	< 0.001
Slow early response^e^	3.32 (2.19-5.03)	< 0.001	4.21 (2.66-6.66)	< 0.001
Poor prednisone response^f^	1.12 (0.77-1.64)	0.556	0.91 (0.57-1.45)	0.686
Initial WBC count ≥100,000^g^	1.39 (0.96-2.02)	0.078	1.50 (0.96-2.32)	0.074

^a^ Hazard ratios (HR) are given as indicated with the corresponding 95% confidence intervals (95% CI), all patients of the study cohort with complete information were included in this analyses (*n* = 1518 of 1547)

^b^ HR compared patients with high bilirubin serum levels ≥ grade 3 of the Common Toxicity Criteria of the National Cancer Institute version 2 (CTC) with patients presenting normal levels or moderate levels

^c^ HR compared patients with severe alanine (ALT) or aspartate (AST) transaminase activity levels ≥ CTC grade 4 with patients presenting normal or moderately elevated levels

^d^ Minimal residual disease (MRD) standard risk, negative on treatment days 33 and 78; MRD high risk, leukemic cell load ≥5 × 10-4 on treatment day 78; all other results MRD intermediate risk. HR compared with the other respective MRD groups

^e^ MRD ≥5 × 10-4 on treatment day 33 and positivity of < 5 × 10-4 on treatment day 78. HR compared with MRD intermediate-risk patients with no slow early response

^f^ Leukemic blasts ≥1000/μL in the peripheral blood on treatment day 8. HR compared with patients with ≥1000/μL leukemic blasts

^g^ HR compared patients with a white blood cell (WBC) count at diagnosis ≥100,000 /μL with patients presenting WBC counts < 100,000 /μL

### Characteristics of the GWAS discovery cohort

The finally pruned GWAS discovery cohort included 650 of 1547 patients with available genome-wide genotyping information (Suppl. Table 3). Both the distribution of hyperbilirubinemia and the clinical characteristics were comparable to those of the entire study population (Table 1 and Suppl. Tables 3 and 4).

Of the 650 patients in this discovery cohort, 215 (33%) patients had normal and 435 (67%) had increased bilirubin levels during induction/consolidation: 367 (56.5%) patients demonstrated moderate hyperbilirubinemia (grades 1-2; 313 during induction, 248 during consolidation, 159 in both phases) and 68 (10.5%) had high hyperbilirubinemia (grades 3-4; 59 reports during induction, 21 during consolidation, 12 in both phases). Pre-treatment hyperbilirubinemia at diagnosis was rare, but more frequent among patients developing chemotherapy-related hyperbilirubinemia during induction/consolidation compared to those not (7.5% (19/252) vs. 1.7% (2/116), *P =* 0.025). Considering the entire course of therapy, 91 patients (14.0%) had high hyperbilirubinemia in at least one treatment element.

### Genome-wide association study

When comparing the 435 patients with hyperbilirubinemia (grades 1-4) during induction/consolidation to the 215 patients with normal bilirubin levels, the five loci most associated with this phenotype were the *UGT1A* gene cluster, *MARK2P5*, *SULF2*, *MIR924HG* and *USH2A* (Suppl. Table 5). The strongest associations were observed for variants residing in the *UGT1A* (*UDP glucuronosyltransferase family 1 member A)* locus at 2q37. The only variant reaching near genome-wide significance (OR = 2.1, 95% CI = 1.6-2.7, *P* = 7.3 × 10^− 8^), rs6744284 was also the index SNV of a 189 kb region of high linkage disequilibrium (LD). Information on the complex *UGT1A* cluster with its overlapping genes and results of imputation are presented in supplementary material (Suppl. Fig. 3, Suppl. Tables 6 and 7).

To examine whether inclusion of age and immunophenotype would influence allelic association, we compared results from crude and adjusted logistic regression analyses. We did not detect any differences in conferred risk for the variant rs6744284 T allele with reference to the wild-type C allele (unadjusted allelic OR = 2.1, 95% CI = 1.6-2.7, *P* = 1.8 × 10^− 7^; adjusted OR = 2.1, 95% CI = 1.6-2.8, *P* = 1.2 × 10^− 7^) (Suppl. Table 5).

**Fig. 2:**
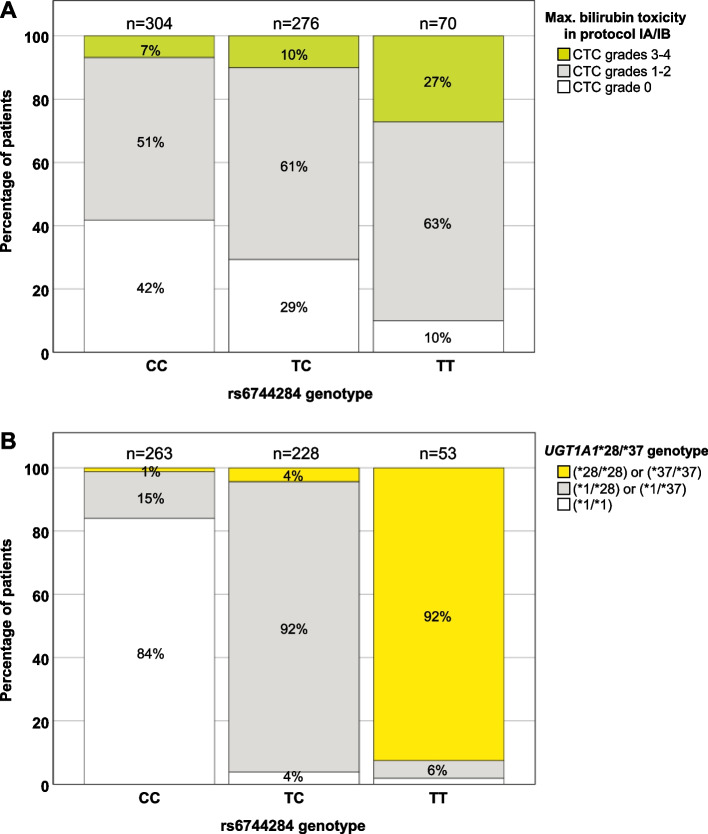
Frequency of rs6744284 genotype by bilirubin toxicity grading in induction/consolidation (protocols IA/IB) treatment according to the Common Toxicity Criteria of the National Cancer Institute, version 2 (CTC) (**A**), and by UGT1A1*28/*37 genotype (**B**). The number of patients (n) for each rs6744284 genotype is given above the columns. **A** analysis based on 650 patients of the discovery cohort; (**B**) based on a subset of 544 patients subsequently genotyped for *UGT1A*1*28/*37 depending on availability of DNA

The genotypic association of rs6744284 with frequency and risk of hyperbilirubinemia during induction/consolidation increased stepwise (Table 3). Compared to wild-type patients (CC; 58% with hyperbilirubinemia grades 1-4), heterozygotes (TC; 71% with hyperbilirubinemia grades 1-4) demonstrated a 1.7-fold higher risk of hyperbilirubinemia, while homozygosity for the T allele (TT; 90% with hyperbilirubinemia grades 1-4) conferred an OR of 6.5 (95% CI = 2.9-14.6, *P* = 7.0 × 10^− 6^) (Fig. 2A and Table 3). Inclusion of age and immunophenotype as covariates or as stratifying variables did not significantly alter these results (Suppl. Tables 7, 8 and 9). Notably, TT-homozygotes were at particular risk of developing high hyperbilirubinemia (grades 3-4, OR with reference to CC genotype 16.4, 95% CI = 6.1-43.8, *P* = 2 × 10^− 8^).

**Table 3:** Association between rs6744284 genotype and risk of hyperbilirubinemia during different treatment elements

Cohort^**a**^	Treatment element	Study individuals^**b**^ n (%)			Frequency of cases and controls per rs6744284 genotype^**c**^ n (%)						Risk in heterozygotes and homozygotes^**d**^			
					TT		TC		CC					
		Controls	Cases	Total	Controls	Cases	Controls	Cases	Controls	Cases	OR_**TC**_(95% CI)	***P***	OR_**TT**_ (95% CI)	***P***
1	Protocol IA/IB	215(33%)	435(67%)	650	7(10%)	63(90%)	81(29%)	195(71%)	127(42%)	177(58%)	1.73(1.22-2.44)	0.002	6.46(2.86-14.57)	< 0.001
1	Protocol IA	254(40%)	372(60%)	626	12(18%)	54(82%)	95(36%)	171(64%)	147(50%)	147(50%)	1.80(1.28-2.53)	< 0.001	4.50(2.31-8.76)	< 0.001
1	Protocol IB	352(67%)	269(43%)	621	17(25%)	50(75%)	131(51%)	127(49%)	204(69%)	92(31%)	2.15(1.52-3.04)	< 0.001	6.52(3.57-11.92)	< 0.001
1	Protocol M	302(61%)	190(39%)	492	17(34%)	33(66%)	126(61%)	82(39%)	159(68%)	75(32%)	1.38(0.93-2.04)	0.106	4.12(2.16-7.85)	< 0.001
1	Protocol II/III	349(76%)	110(24%)	459	16 (37%)	27(63%)	139(75%)	47(25%)	194(84%)	36(16%)	1.82(1.12-2.96)	0.015	9.09(4.46-18.56)	< 0.001
1	HR blocks	44(41%)	63 (59%)	107	1 (8%)	12(92%)	15(34%)	29(66%)	28(56%)	22(44%)	2.46(1.07-5.68)	0.035	15.27(1.84-126.61)	0.012
2	Protocol IA/IB	79(35%)	145 (65%)	224	3 (13%)	20(87%)	27(28%)	71(72%)	49(48%)	54(52%)	2.39(1.33-4.30)	0.004	6.05(1.69-21.62)	0.006
1 + 2^e^	Protocol IA/IB	294(34%)	580(66%)	874	10(3%)	108(37%)	176(60%)	83(14%)	266(46%)	231(40%)	1.88(1.39-2.53)	< 0.001	6.32(3.19-12.54)	< 0.001

^a^ Association of rs6744284 genotypes with hyperbilirubinemia was tested in two independent cohorts derived from the AIEOP-BFM ALL 2000 trial: 650 patients of the derivation cohort (1) were analyzed according to the maximum bilirubin levels detected during protocols I (induction (IA) and consolidation (IB), M (extracompartment therapy), II or III (late intensification) and HR (high-risk block treatment). The 224 patients of the replication cohort (2) were analyzed based on the bilirubin levels detected during induction/consolidation (protocols IA/IB), only

^b^ As in the initial genome-wide analysis performed for induction/consolidation (protocols IA/IB), individuals of the other protocol elements demonstrating hyperbilirubinemia(CTC grades 1-4) were considered as cases and compared to control patients with normal bilirubin levels (controls, CTC grade 0). Toxicity grading was according to the Common Toxicity Criteria (CTC) of the National Cancer Institute, version 2

^c^ The amount of controls and cases per specified rs6744284 genotype is given as indicated: homozygotes for the risk/minor allele (TT), heterozygotes (TC) and homozygotes for the major allele (CC)

^d^ Genotypic association was analyzed using binary logistic regression. Odds ratios (OR) and corresponding 95% confidence intervals (CI) are listed for heterozygous (TC) and homozygous genotypes (TT)

^e^ Combining both datasets the allelic association of rs6744284 with hyperbilirubinemia during protocol IA/IB reached genomewide significance (OR, 2.1, CI, 1.70-2.68, *P*(*X*^*2*^) = 5.75 × 10^−11^)

### Independent replication analysis

We performed independent replication analyses in a cohort of 224 *ETV6-RUNX1*-rearranged pediatric ALL patients (Suppl. Table 10), results of which supported our initial GWAS findings. The allelic OR for hyperbilirubinemia (grades 1-4) during induction/consolidation conferred by the variant rs6744284 T allele versus the wild-type C allele was 2.3 (95% CI = 1.5-3.7, *P* = 2.4 × 10^− 4^). Genotypic OR in comparison to wild-type patients (CC) were 2.4 (95% CI = 1.3-4.3, *P* = 3.8 × 10^− 3^) for heterozygotes (TC) and 6.1 (95% CI = 1.7-21.6, *P* = 5.6 × 10^− 3^) for homozygous variant patients (TT) (Table 3). Similar to our findings in the GWAS discovery cohort, patients possessing the rs6744284 TT genotype were at particular risk of high hyperbilirubinemia (OR with reference to CC genotype 13.6, 95% CI = 2.6-71.8, *P* = 0.002).

Association testing in the combined discovery and replication cohorts resulted in genome-wide significance. Compared to the rs6744284 wild-type allele, presence of the T allele was associated with an OR of 2.1 (CI = 1.7-2.7) for hyperbilirubinemia (grades 1-4) during induction/consolidation at a significance level of *P* = 5.7 × 10^− 11^.

### *UGT1A* rs6744284 genotype in subsequent treatment elements

Similar to initial findings, we observed that the rs6744284 TT genotype was also strongly associated with hyperbilirubinemia during extracompartment therapy (Protocol M, OR = 4.1, 95% CI = 2.2-7.9; *P* < 0.001), re-intensification (Protocols II and III, OR = 9.1, 95% CI = 4.5-18.6, *P* < 0.001) and high-risk (HR) block elements (OR = 15.3, 95% CI = 1.8-126.6; *P* = 0.012) (Table 3, Suppl. Fig. 4). Thus, the effect of rs6744284 on risk of hyperbilirubinemia was not limited to early chemotherapy, but was generalizable to all intensive treatment phases for pediatric ALL.

### *UGT1A* rs6744284 and Gilbert’s syndrome-associated variants

The UGT1A enzyme family is crucial for bilirubin glucuronidation and related impairing genetic alterations form the mechanistic basis of the Gilbert’s syndrome (GS) [18–20]. Therefore, we genotyped the GS-related functional genetic variations UGT1A1*28 and *37 [21, 22] in 544 (84%) patients of our discovery cohort with available remission DNA (Suppl. Table 11). Comparable to rs6744284, we observed a strong association with hyperbilirubinemia: the allelic OR for *28/*37 vs. wild-type (*1) was 1.9 (95% CI = 1.4-2.5, *P =* 5.0 × 10^− 6^). Genotype-based analyses demonstrated a stepwise increase of frequency and risk of hyperbilirubinemia for the variant alleles. Out of 544 patients 62 (11%) were homozygous for either UGT1A1*28/*28 or *37/*37 – this cannot be differentiated by our assay – and had the highest rate and risk of hyperbilirubinemia (89% compared to 58% for *1/*1; OR in comparison to *1/*1 5.8; 95% CI = 2.5-13.3; *P* = 3.3 × 10^− 5^) (Suppl. Table 12). Homozygous variant patients were at particular risk of developing high hyperbilirubinemia (grades 3-4, OR = 12.4, 95% CI = 4.4-34.8, *P* = 1.9 × 10^− 6^). The strong interrelationship of rs6744284 with UGT1A1*28/*37 is depicted in Fig. 2B. Extended haplotype analyses including eight additional GS-related variants further documented a strong association with rs6744284 (see Suppl. Table 13 and related additional information). Of note, none of the GS-related variants showed a stronger association with hyperbilirubinemia than rs6744284.

### Hyperbilirubinemia, transaminase levels, rs6744284 genotype, treatment delay and outcome in the GWAS discovery cohort (*n* = 650)

Similar to the patients of the entire study population (*n* = 1547), patients in our discovery cohort with high hyperbilirubinemia during induction/consolidation tended to take 2 days longer to complete consolidation (*P* = 0.072) (for details see Suppl. Information, page 25). Of interest, we did not observe significant differences between rs6744284 genotypes: 88 days for TT vs. 89 days for CT and 90 days for CC (*P* = 0.122).

**Fig. 3:**
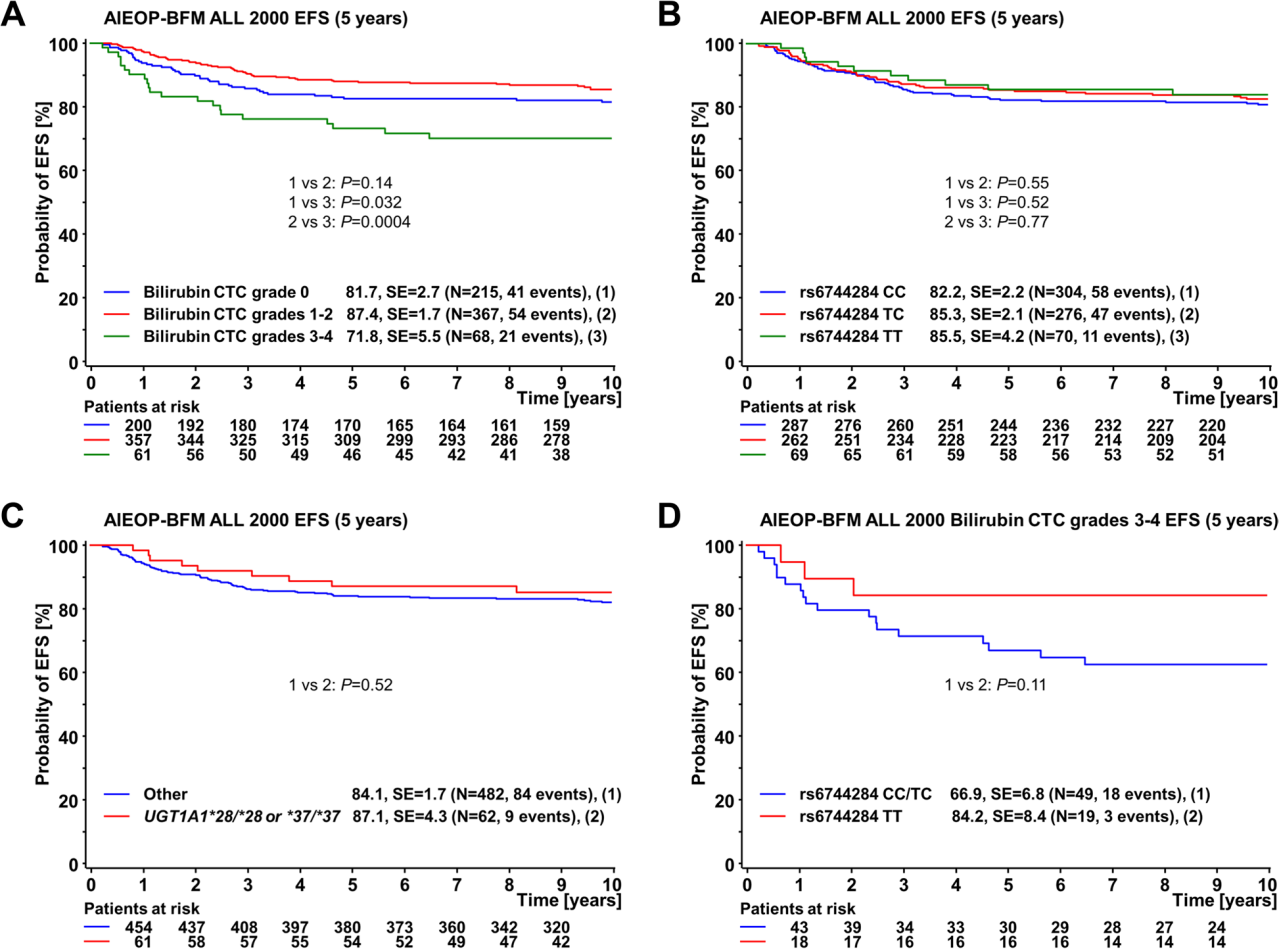
Event-free survival (EFS) at 5 years in ALL patients from the discovery cohort according to (**A**) maximum total bilirubin toxicity grade during induction/consolidation (protocols IA/IB); (**B**) rs6744284 genotype (CC, TC, TT); and (**C**) homozygosity for the UGT1A1*28 or *37 allele. **D** illustrates the EFS by rs6744284 genotype (CC/TC and TT) restricted to patients with high bilirubinemia (*n* = 68; grades 3 and 4). Bilirubin toxicity grading was according to the Common Toxicity Criteria (CTC) of the National Cancer Institute, version 2; standard error (SE) and the number of included individuals are indicated for each category

**Fig. 4:**
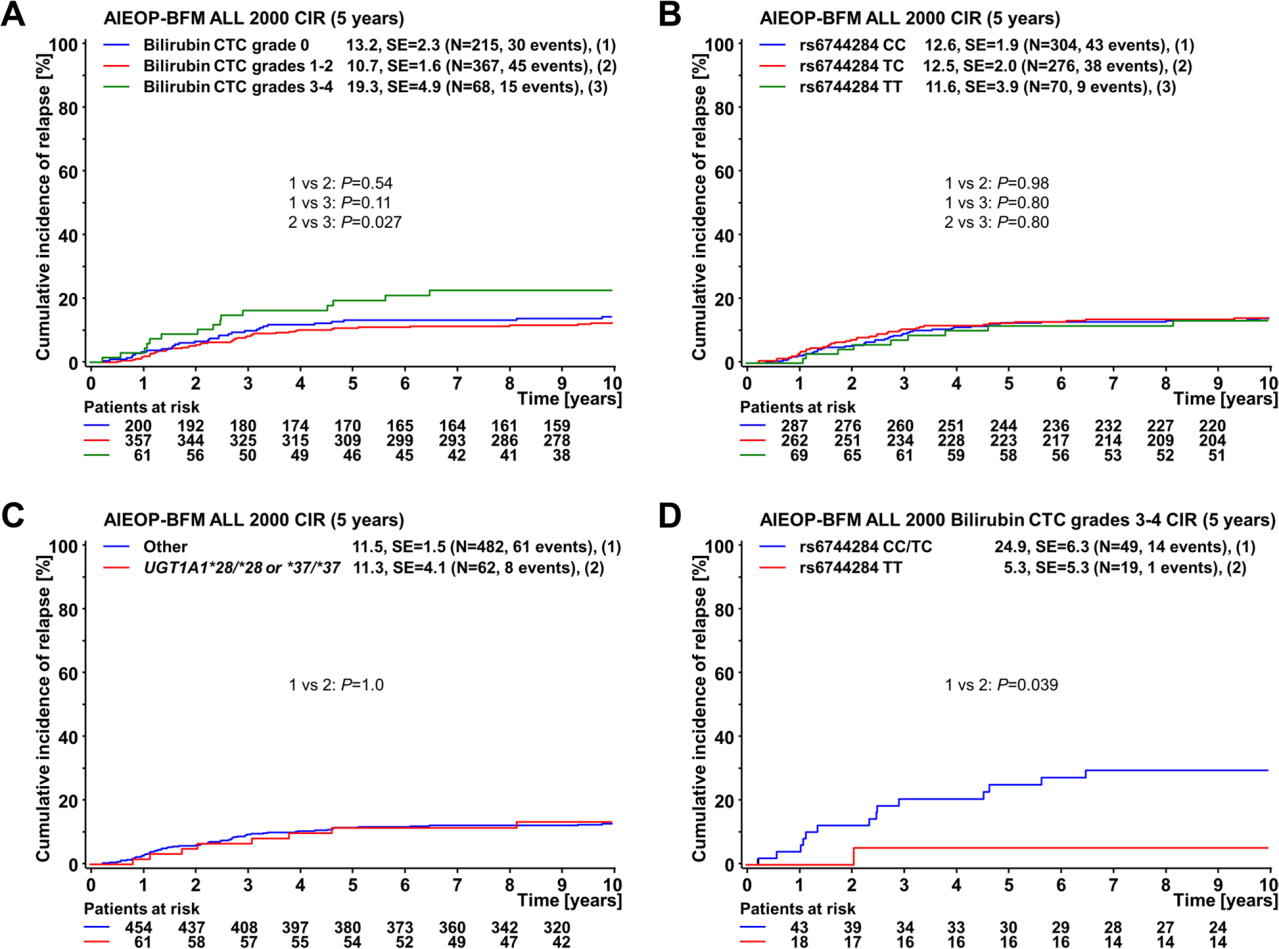
Cumulative incidence of relapse (CIR) at 5 years in ALL patients from the discovery cohort according to (**A**) maximum total bilirubin toxicity grade during induction/consolidation (protocols IA/IB); (**B**) rs6744284 genotype (CC, TC, TT); and (**C**) homozygosity for the UGT1A1*28 or *37 allele. **D** shows the effect of the rs6744284 genotype (CC/TC and TT) on CIR in the group of patients with high bilirubin levels (*n* = 68; grades 3 and 4). Bilirubin toxicity grading was according to the Common Toxicity Criteria (CTC) of the National Cancer Institute, version 2; standard error (SE) and the number of included individuals are indicated for each category

Consistent with the results obtained for the entire study cohort, outcome analyses of the discovery cohort showed that high hyperbilirubinemia during induction/consolidation was associated with a poor 5-year EFS of 71.8 ± 5%, compared to 87.4 ± 2% and 81.7 ± 3% in patients with moderate and without hyperbilirubinemia, respectively (Fig. 3A). The corresponding 5-year CIR were 19.3 ± 5% for high hyperbilirubinemia, 10.7 ± 2% for moderate, and 13.2 ± 2% for no hyperbilirubinemia (Fig. 4A). Although rs6744284 was strongly associated with high hyperbilirubinemia and the proportion of patients with TT genotype among those with high hyperbilirubinemia was 28% (19/68), there were no differences between rs6744284 genotypes related to EFS or CIR (Figs. 3B and 4B) in the discovery cohort. However, within high hyperbilirubinemic patients those carrying the TT genotype had a better EFS (84.2 ± 8% vs 66.9 ± 7%, *P* = 0.110) and a lower CIR (5.3 ± 5% vs 24.9 ± 6%, *P* = 0.039) at 5 years compared to the remaining genotypes (TC, CC) (Figs. 3D and 4D).

In the GWAS discovery cohort 604 (93%) patients were observed with elevated hepatic transaminase levels (grades 1-4) during induction/consolidation (Suppl. Table 3), 315 of which demonstrated high grades 3-4. Similar to the results obtained for the 1443 patients in the entire study cohort with available information, transaminase levels were positively associated with hyperbilirubinemia. Especially patients with high hyperbilirubinemia were at increased risk for concurrent high transaminase levels compared to the remaining patients (75% vs. 41%, OR = 16.8, 95% CI = 2.2-127.1, *P* = 0.006) (Suppl. Table 14). Of importance, transaminasemia was not associated with rs6744284 genotype (*P* = 0.74). Five-year EFS and CIR did not differ between patients with no (grade 0), moderate (grades 1-2) or high (grades 3-4) transaminasemia during induction/consolidation (Suppl. Fig. 5A and B).

Patients with high hyperbilirubinemia and concurrent high transaminasemia tended to have a higher 5-year EFS of 74.3 ± 6% compared to 64.7 ± 12% in patients with high hyperbilirubinemia accompanied by moderate or no transaminasemia (grades 0-2, *P* = 0.480; Suppl. Fig. 5C). Corresponding CIR were 21.8 ± 6% and 11.8 ± 8% (*P* = 0.260, Suppl. Fig. 5D).

Multivariate analyses including established prognostic factors in AIEOP-BFM trials identified high hyperbilirubinemia as an independent predictor of outcome, while rs6744284 TT genotype demonstrated only a tentative protective effect in these analyses (Table 4). However, in multivariate analysis restricted to patients with prognostically unfavorable high hyperbilirubinemia in induction/consolidation, the rs6744284 TT genotype was associated with a statistically significant 14-fold lower relapse-risk compared to rs6744284 wild-type or heterozygous variant patients (CC or TC) (Suppl. Table 15).

**Table 4:** Estimated hazard ratios^a^ from the multivariable Cox proportional model on event-free survival and hazard of relapse in patients of the discovery cohort

Variable	Event^b^		Relapse	
	Hazard Ratio (95% CI)	***P(X***^***2***^***)***	Hazard Ratio (95% CI)	***P(X***^***2***^***)***
rs6744284 TT^c^	0.56 (0.27-1.13)	0.103	0.40 (0.09-1.84)	0.240
Bilirubin CTC grades 3-4^d^	2.67 (1.59-4.48)	< 0.001	4.61 (1.71-12.42)	0.003
ALT/AST CTC grade 4^e^	1.17 (0.60-2.27)	0.643	0.43 (0.06-3.29)	0.417
MRD standard-risk^f^	0.94 (0.57-1.54)	0.798	1.10 (0.37-3.24)	0.870
MRD high-risk^f^	4.52 (2.73-7.48)	< 0.001	6.52 (2.41-17.66)	< 0.001
Slow early response^g^	2.60 (1.35-5.00)	0.004	2.21 (0.48-10.17)	0.311
Poor prednisone response^h^	1.38 (0.82-2.32)	0.223	2.09 (0.77-5.64)	0.148
Initial WBC count ≥100,000/μL^i^	1.48 (0.92-2.39)	0.109	1.18 (0.43-3.22)	0.750

^a^ Hazard ratios (HR) are given as indicated with the corresponding 95% confidence intervals (CI), all patients of the discovery cohort with complete information were included (*n* = 642) in this analyses

^b^ Events were resistance to therapy (non-response), relapse, secondary neoplasm or death from any cause. Failure to achieve remission due to early death or non-response was considered as event at time zero

^c^ Compared to rs6744284 wild-type (CC) or heterozygous (TC) genotypes

^d^ Compared to bilirubin grades 0 to 2 of the Common Toxicity Criteria of the National Cancer Institute version 2 (CTC)

^e^ Compared to CTC grades 0 to 3 of alanine (ALT) or aspartate (AST) transaminase serum levels

^f^ Minimal residual disease (MRD) standard-risk patients were negative on treatment days 33 and 78; MRD high-risk patients had a leukemic cell load ≥5 × 10^−4^ on treatment day 78; all other results were classified MRD intermediate-risk. MRD standard- or high-risk compared with MRD intermediate-risk

^g^ HR compared with MRD intermediate-risk patients with no slow early response

^h^ Leukemic blasts ≥1000/μL in the peripheral blood on treatment day 8. HR compared to patients with < 1000/μL leukemic blasts (prednisone good responders)

^i^ White blood cell (WBC) count at diagnosis ≥100,000 /μL. HR compared to patients with WBC counts < 100,000 /μL

## Discussion

Routine hepatotoxicity monitoring in clinical trials for pediatric ALL is typically performed by grading of elevated bilirubin and liver transaminase levels as absent, mild, moderate, severe or life-threatening/fatal according to the NCI CTC criteria. Although the evaluation of laboratory values for hepatotoxicity is common practice, it can be debated how precise such measurements reflect liver dysfunction. To enhance the phenotypic characterization in context with abnormal laboratory values, alternative classifications integrate additional clinical information (e.g., coagulopathy, impairment of liver function-dependent organs) [23]. Similarly, genetic biomarkers hold the potential to aid strategies directed at improved evaluation of hepatotoxicity. Nonetheless, only a few are currently used in clinical routine to guide a genotype-adapted dosing of specific chemotherapeutic agents and thereby reduce adverse reactions (e.g., *TPMT* with thiopurines [24]*, UGT1A1* with irinotecan [25, 26]). In the present study, we applied an unbiased genome-wide approach and identified genetic variation in the *UGT1A* gene cluster as a major contributor to hyperbilirubinemia associated with chemotherapy for pediatric ALL.

Genetic variation in *UGT1A* is well-established to affect enzymatic glucuronidation activity and to modulate the metabolism of endogenous metabolites as well as multiple xenobiotics [27–35]. Out of nine functional isoforms, only *UGT1A1* is relevant for bilirubin glucuronidation [31, 36]. Various functional *UGT1A1* variants result in partial or complete reduction of enzymatic activity and determine the phenotype of heritable diseases of bilirubin metabolism [31, 36, 37]. The most common genetic cause for reduced bilirubin conjugation is an insertion polymorphism in the TATA box of *UGT1A1* – the (TA)7 variant allele UGT1A1*28; homozygosity for this allele confers a reduced transcriptional activity of 18 to 33% [22, 38], corresponding to the residual glucuronidation activity of ~ 30% determined in patients with Gilbert’s syndrome (GS) [39, 40]. While other associated risk alleles have been described, e.g. UGT1A1*6 (rs4148323) in Asian populations [41], UGT1A1*28 is by far the most common cause for the Gilbert’s syndrome in Caucasians and African Americans [22, 37]. Our lead SNV, rs6744284, was closely correlated to all GS-related variants assessable in our investigational setting – including UGT1A1*28 – and was the best predictor of hyperbilirubinemia in our patients. These findings are concordant with reports on multi-SNV haplotypes of *UGT1A* involved in impaired glucuronidation associated with GS [20, 42] and support a potential diagnostic role for the simple assessment of rs6744284.

With relevance to cancer treatment, UGT1A1*28 or UGT1A1*6 homozygotes are low metabolizers of irinotecan and have an increased risk of severe neutropenia, requiring preventive dose adjustments [25, 43]. Likewise, UGT1A*28 homozygotes are recommended to receive reduced doses of belinostat [44, 45]. The *UGT1A1* genotype also influences the pharmacokinetics of other glucuronidation-dependent drugs – including several ones used in ALL treatment (e.g., methotrexate, etoposide, and cyclophosphamide) [28, 32, 33, 35, 46, 47]. For example, low UGT1A1 activity was associated with higher plasma methotrexate and bilirubin levels, suggestive of competitive interactions between the three [7, 48]. Similar to our study, these former investigations in the field of pediatric ALL showed that patients with GS were prone to hyperbilirubinemia throughout all treatment phases [7, 48].

One of the previous studies demonstrated that despite higher bilirubin levels, ALL patients with GS did not experience significant treatment modifications, including delays, or worse therapy outcomes [48], which is in line with our findings. These observations may explain why universal screening for GS in all patients diagnosed with ALL is not routinely performed so far. Although it was recommended previously to screen at least ALL patients with hyperbilirubinemia for GS [48], common standard recommendations agreed on between international ALL trial consortia do not exist, and may be promoted through the Ponte di Legno initiative [23].

In our study, high hyperbilirubinemia was an independent prognostic factor negatively affecting EFS and CIR of patients treated on a modern risk-adapted BFM protocol. To our knowledge, this is the first report from a large pediatric ALL cohort receiving relatively homogenous therapy demonstrating an effect of hyperbilirubinemia on long-term treatment outcome. Importantly, the negative prognostic impact was not determined in patients who demonstrated high hyperbilirubinemia and were homozygous for the variant T allele of our lead SNV rs6744284. This observation could have direct clinical implications by helping to differentiate hyperbilirubinemia conferring a negative prognostic impact from hyperbilirubinemia of a less severe clinical phenotype. Moreover, our findings imply that patients with phenotypically relevant genetic variation in *UGT1A/GS* can be spared from experiencing treatment modifications, as it was suggested previously [48, 49]. Besides replication in other independent clinically and genetically comprehensively characterized cohorts, functional studies are particularly required to augment our knowledge of hyperbilirubinemia as a treatment-related toxicity in pediatric ALL and to elucidate involved pathomechanisms.

Despite interesting perspectives, there are several limitations associated with our study: 1) We were only able to study total serum bilirubin levels. However, a separate analysis of unconjugated and conjugated bilirubin will be important for an improved understanding of therapy-related hyperbilirubinemia and its prognostic value. 2) No data on additional intake (e.g., vitamin B complex, ursodiol) and/or pharmacokinetics of glucuronidation dependent drugs (e.g., methotrexate), potentially confounding our observations, were available to us. 3) Selection bias for inclusion in our GWAS and/or replication cohorts is immanent to a clinical investigation depending on availability of reported hepatotoxicity and biological material. 4) The clinical importance of our findings could be enhanced by systematic collection of information on the potential long-term burden of hepatotoxicity. 5) Finally, the generalizability of our findings to other therapy protocols for pediatric ALL is limited due to differences in medication and timing between them. All of these issues need to be addressed in future investigations and will help to resolve the limitations of our current observations.

## Conclusions

High hyperbilirubinemia acted as an independent prognostic factor of therapy outcome in pediatric ALL patients treated on the AIEOP-BFM ALL 2000 protocol. Further, the rs6744284 genotype reliably predicted hyperbilirubinemia throughout all intensive treatment phases. Thus, both assessment of hyperbilirubinemia and *UGT1A* genotyping will be useful for complementing toxicity risk profiling and optimizing risk-adapted therapeutic strategies for pediatric ALL.

## Supplementary Information

**Additional file 1: Suppl. Table 1.** Treatment details of protocol AIEOP-BFM ALL 2000. **Suppl. Table 2.** Clinical characteristics of the patients in the study cohort by severity of bilirubin toxicity during induction/consolidation (protocols IA/IB, *n* = 1547). **Suppl. Table 3.** Characteristics of the patients in the GWAS discovery cohort by serum bilirubin levels during induction/consolidation (*n* = 650). **Suppl. Table 4.** Characteristics of the patients in the GWAS discovery cohort compared to all patients of the study cohort with toxicity information. **Suppl. Table 5.** Summary of genome-wide association analysis for therapy-related hyperbilirubinemia during induction/consolidation (protocols IA/IB). **Suppl. Table 6.** Allelic association of hyperbilirubinemia phenotype with the 20 most strongly associated variants around rs6744284 resulting from genotype imputation. **Suppl. Table 7.** Genotypic association between hyperbilirubinemia phenotype and the 20 most strongly associated SNV around rs6744284 after genotype imputation. **Suppl. Table 8.** Adjusted genotypic association of rs6744284 with hyperbilirubinemia during protocols IA/IB and later therapeutic elements, including age and immunophenotype as covariates. **Suppl. Table 9.** Genotypic association of rs6744284 with hyperbilirubinemia phenotype stratified for potential effect modifiers. **Suppl. Table 10.** Characteristics of acute lymphoblastic leukemia (ALL) patients included in the replication cohort (*n* = 224). **Suppl. Table 11.** Characteristics of acute lymphoblastic leukemia (ALL) patients included in subsequent UGT1A1*28/*37 genotyping (*n* = 544). **Suppl. Table 12.** Association of identified risk loci and known Gilbert’s syndrome related variants with hyperbilirubinemia. **Suppl. Table 13.** Correlation of rs6744284 with imputed top SNV and *UGT1A* variations related to hyperbilirubinemia and the Gilbert’s syndrome. Suppl. information on the correlation analysis of known Gilbert’s syndrome (GS) related variations with rs6744284. Suppl. information on the impact of hyperbilirubinemia on therapy delays in the discovery cohort. **Suppl. Table 14.** Clinical characteristics of the acute lymphoblastic leukemia (ALL) patients of the discovery cohort according to the severity of bilirubin toxicity during induction/consolidation (protocols IA/IB, *n* = 650). **Suppl. Table 15.** Estimated hazard ratios from the multivariable Cox proportional model on the hazard of relapse in patients with high hyperbilirubinemia (≥CTC grade 3) during induction and/or consolidation (*n* = 68). **Suppl. Fig. 1.** Consolidated Standards of Reporting Trials (CONSORT) diagram of inclusion criteria for the study population (*N* = 1547). **Suppl. Fig. 2.** Estimated 5-year event-free survival (EFS) and cumulative incidence of relapse (CIR) at 5 years in the study cohort by the maximum transaminase levels during protocol IA/IB [%]. **Suppl. Fig. 3.** Regional plot of association results and recombination rates for the identified risk locus in the UGT1A region (2q37). **Suppl. Fig. 4.** Total serum bilirubin levels by treatment element and rs6744284 genotype. **Suppl. Fig. 5.** Estimated 5-year event-free survival (EFS) and cumulative incidence of relapse (CIR) at 5 years in the discovery cohort by the maximum transaminase levels during protocol IA/IB [%].

## Data Availability

All data generated or analysed during this study are included in this published article and its supplementary information files.

## Abbreviations

AIEOP-BFM ALL study group: Associazione Italiana Ematologia Oncologia Pediatrica - Berlin Frankfurt-Muenster Acute Lymphoblastic Leukemia study group
ALL: Acute lymphoblasic leukemia
CI: Confidence Interval
CIR: Cumulative incidence of relapse
CNS: Central nervous system
CSF: Cerebrospinal fluid
CTC: Common toxicity criteria of the NCI
d: Day
EFS: Event free survival
*ERG*: ETS Transcription Factor ERG
*ETV6*: ETS Variant Transcription Factor 6
GS: Gilbert’s syndrome
GWAS: Genomwide Association Study
Gy: Gray
HR: High risk
IR: Intermediate risk
IT: Intrathecal administration
IU/m^2^: International Unit per Square Meter
IV: Intravenous infusion
kb: Kilobase
LD: Linkage disequilibrium
*MARK2P5*: Microtubule Affinity Regulating Kinase 2 Pseudogene 5
μL: Microliter (1 μL = 1 × 10^− 6^ l)
mg: Miligram (1 mg =1 × 10^− 3^ g)
*MIR924HG*: MIR924 Host Gene
MRD: Minimal residual disease
MTX: methotrexate
NCI: National Cancer Institute
OR: Odds Ratio
PI: Intravenous push
PO: Oral administration
*RUNX1*: RUNX family transcription factor 1
SNV: Single nucleotide variant
SR: Standard risk
*SULF2*: Sulfatase 2
Suppl.: Supplementary
*TPMT*: Thiopurine methyltransferase
UDP: Uridine 5′-diphospho-glucuronosyltransferase
*UGT1A*: UDP Glucuronosyltransferase Family 1 Member A
UNL: Upper normal limit /upper limit of normal
*USH2A*: Usherin
WBC: white blood cell
