# Supplementary material for: Chemotherapy-related hyperbilirubinemia in pediatric acute lymphoblastic leukemia: a genome-wide association study from the AIEOP-BFM ALL study group
Source: J Exp Clin Cancer Res. 2023 Jan 13;42:21. doi: 10.1186/s13046-022-02585-x (PMC9838013; doi:10.1186/s13046-022-02585-x)
Supplement: Supplementary file 1 — Additional file 1: Suppl. Table 1. Treatment details of protocol AIEOP-BFM ALL 2000. Suppl. Table 2. Clinical characteristics of the patients in the study cohort by severity of bilirubin toxicity during induction/consolidation (protocols IA/IB, n = 1547). Suppl. Table 3. Characteristics of the patients in the GWAS discovery cohort by serum bilirubin levels during induction/consolidation (n = 650). Suppl. Table 4. Characteristics of the patients in the GWAS discovery cohort compared to all patients of the study cohort with toxicity information. Suppl. Table 5. Summary of genome-wide association analysis for therapy-related hyperbilirubinemia during induction/consolidation (protocols IA/IB). Suppl. Table 6. Allelic association of hyperbilirubinemia phenotype with the 20 most strongly associated variants around rs6744284 resulting from genotype imputation. Suppl. Table 7. Genotypic association between hyperbilirubinemia phenotype and the 20 most strongly associated SNV around rs6744284 after genotype imputation. Suppl. Table 8. Adjusted genotypic association of rs6744284 with hyperbilirubinemia during protocols IA/IB and later therapeutic elements, including age and immunophenotype as covariates. Suppl. Table 9. Genotypic association of rs6744284 with hyperbilirubinemia phenotype stratified for potential effect modifiers. Suppl. Table 10. Characteristics of acute lymphoblastic leukemia (ALL) patients included in the replication cohort (n = 224). Suppl. Table 11. Characteristics of acute lymphoblastic leukemia (ALL) patients included in subsequent UGT1A1*28/*37 genotyping (n = 544). Suppl. Table 12. Association of identified risk loci and known Gilbert’s syndrome related variants with hyperbilirubinemia. Suppl. Table 13. Correlation of rs6744284 with imputed top SNV and UGT1A variations related to hyperbilirubinemia and the Gilbert’s syndrome. Suppl. information on the correlation analysis of known Gilbert’s syndrome (GS) related variations with rs6744284. Suppl. inform [file 13046_2022_2585_MOESM1_ESM.pdf]

Supplementary information to

Chemotherapy-related hyperbilirubinemia in pediatric acute lymphoblastic leukemia: a genome-wide association study from the AIEOP-BFM ALL Study Group

Stefanie V. Junk[1], Elke Schaeffeler[2], Martin Zimmermann[1], Anja Möricke[3], Rita Beier[1], Peter Schütte[1], Birthe Fedders[3], Julia Alten[3], Laura Hinze[1], Norman Klein[1], Andreas Kulozik[4], Martina U. Muckenthaler[4], Rolf Koehler[5] Arndt Borkhardt[6], Jayaram Vijayakrishnan[7], David Ellinghaus[3], Michael Forster[8], Andre Franke[8], Astrid Wintering[1], Christian P. Kratz[1], Martin Schrappe[3], Matthias Schwab[2,9,10,11], Richard S. Houlston[7], Gunnar Cario[3], and Martin Stanulla[1,§]

1 Pediatric Hematology and Oncology, Hannover Medical School, Hannover, Germany
2 Margarete-Fischer-Bosch Institute of Clinical Pharmacology, Stuttgart, Germany
3 Department of Pediatrics, University Hospital Schleswig-Holstein, Kiel, Germany
4 Department of Pediatric Hematology, Oncology and Immunology, University of Heidelberg, Heidelberg, Germany
5 Department of Human Genetics, University of Heidelberg, Heidelberg, Germany
6 Clinic for Pediatric Oncology, Hematology, and Clinical Immunology, Medical Faculty, Heinrich-Heine-University, Düsseldorf, Germany
7 Division of Genetics and Epidemiology, The Institute of Cancer Research, Sutton, Surrey, United Kingdom
8 Institute of Clinical Molecular Biology, Kiel University, Kiel, Germany
9 Departments of Clinical Pharmacology, and of Biochemistry and Pharmacy, University of Tübingen, Tübingen, Germany
10 Cluster of Excellence iFIT (EXC 2180) „Image-Guided and Functionally Instructed Tumor Therapies“, University of Tübingen, Germany
11 German Cancer Consortium (DKTK) and German Cancer Research Center (DKFZ) Partner Site Tübingen, Tübingen, Germany
§ corresponding author

Supplementary Appendix Content

Supplementary Methods

Supplementary Tables

Suppl. Table 1. Treatment details of protocol AIEOP-BFM ALL 2000.

Suppl. Table 2. Clinical characteristics of the patients in the study cohort by severity of bilirubin toxicity during induction/consolidation (protocols IA/IB, n=1547).

Suppl. Table 3. Characteristics of the patients in the GWAS discovery cohort by serum bilirubin levels during induction/consolidation (n=650).

Suppl. Table 4. Characteristics of the patients in the GWAS discovery cohort compared to all patients of the study cohort with toxicity information.

Suppl. Table 5. Summary of genome-wide association analysis for therapy-related hyperbilirubinemia during induction/consolidation (protocols IA/IB).

Suppl. Table 6. Allelic association of hyperbilirubinemia phenotype with the 20 most strongly associated variants around rs6744284 resulting from genotype imputation

Suppl. Table 7. Genotypic association between hyperbilirubinemia phenotype and the 20 most strongly associated SNV around rs6744284 after genotype imputation.

Suppl. Table 8. Adjusted genotypic association of rs6744284 with hyperbilirubinemia during protocols IA/IB and later therapeutic elements, including age and immunophenotype as covariates.

Suppl. Table 9. Genotypic association of rs6744284 with hyperbilirubinemia phenotype stratified for potential effect modifiers.

Suppl. Table 10. Characteristics of acute lymphoblastic leukemia (ALL) patients included in the replication cohort (n=224).

Suppl. Table 11. Characteristics of acute lymphoblastic leukemia (ALL) patients included in subsequent U*GT1A1**28/*37 genotyping (n=544).

Suppl. Table 12. Association of identified risk loci and known Gilbert's syndrome related variants with hyperbilirubinemia.

Suppl. Table 13. Correlation of rs6744284 with imputed top SNV and *UGT1A* variations related to hyperbilirubinemia and the Gilbert's syndrome.

Suppl. information on the correlation analysis of known Gilbert's syndrome (GS) related variations with rs6744284

Suppl. information on the impact of hyperbilirubinemia on therapy delays in the discovery cohort

Suppl. Table 14. Clinical characteristics of the acute lymphoblastic leukemia (ALL) patients of the discovery cohort according to the severity of bilirubin toxicity during induction/consolidation (protocols IA/IB, n=650).

Suppl. Table 15. Estimated hazard ratios from the multivariable Cox proportional model on the hazard of relapse in patients with high hyperbilirubinemia (≥CTC grade 3) during induction and/or consolidation (n=68).

Supplementary References

Supplementary Figures

Suppl. Figure 1. Consolidated Standards of Reporting Trials (CONSORT) diagram of inclusion criteria for the study population (N=1547).

Suppl. Figure 2. Estimated 5-year event-free survival (EFS) and cumulative incidence of relapse (CIR) at 5 years in the study cohort by the maximum transaminase levels during protocol IA/IB [%].

Suppl. Figure 3. Regional plot of association results and recombination rates for the identified risk locus in the UGT1A region (2q37).

Suppl. Figure 4. Total serum bilirubin levels by treatment element and rs6744284 genotype.

Suppl. Figure 5. Estimated 5-year event-free survival (EFS) and cumulative incidence of relapse (CIR) at 5 years in the discovery cohort by the maximum transaminase levels during protocol IA/IB [%].

Supplementary Methods

Toxicity definitions

As part of the routine safety management, toxicity was assessed for all treatment elements except for interim maintenance and maintenance phases. Considering 17.1 μmol/L as the upper normal limit (UNL), total bilirubin serum levels were graded according to the CTC of the NCI, version 2(1): grade 0: ≤UNL; grade 1: >UNL to 1.5xUNL; grade 2: >1.5x UNL to 3.0x UNL; grade 3: >3.0x UNL to 10.0x UNL and grade 4: >10.0x UNL. Alanine (ALT) and/or aspartate (AST) transaminase levels were also assessed according to the CTC, considering 20 U/L as the UNL: grade 0: ≤UNL; grade 1: >UNL to 2.5xUNL; grade 2: >2.5x UNL to 5.0x UNL; grade 3: >5.0x UNL to 20.0x UNL; grade 4: >20.0x UNL. According to the AIEOP BFM ALL 2000 protocol, upon increased hyperbilirubinemia with or without transaminasemia (≥ grades 3-4 of the CTC) drug administration was sometimes postponed, but complete withdrawals / alterations of therapy were not recommended.

Genome-wide association study

Prior to association testing, we excluded SNV meeting any of the following criteria: call rate <99%, deviation from Hardy-Weinberg equilibrium (P <1x10-5), non-autosomal or location within the major histocompatibility complex region. We excluded 33 patients with a poor genotype call rate (<98%), outlying heterozygosity rate, divergent sex information, cryptical familiar relationship (Proportion IBD>0.2) or non-European ancestry. Ancestry was estimated by multidimensional scaling analysis using the HapMap cohort (phase 2, release 23) as a reference population. After quality control and applying a minimum accepted minor allele frequency of 0.02, 650 patients and 745,895 variants remained in the discovery cohort. The pruned data

set had a total genotyping rate of 0.999 and a low genomic inflation (inflation factor λ=1.004).

Genotype imputation

Imputation for fine-mapping purposes was performed with beagle (version 3.3.2)(2), merging the genotypes of a 5 megabase region around the index SNV from the discovery cohort and the 1000 genomes phase 1 European reference dataset from March 2012 (non-Finnish, GRCh37). Poorly imputed SNV (beagle's allelic r^2<0.3) and those not meeting our quality requirements, mentioned above, were excluded; we visualized SNV with a MAF ≥0.01 of a 500 kb region around the index SNV, using a modified version of the deBakker's R script(3) for regional association plotting.

Statistical analyses

Differences in the distribution of individual parameters among patient subsets were analyzed using the X^2 or Fisher's exact test for categorical and the Kruskal-Wallis test for continuous variables(4). EFS was defined as the time from diagnosis to the date of last follow-up in complete remission (censored time) or first event. Events were resistance to therapy (non-response), relapse, secondary neoplasm or death from any cause. Failure to achieve remission due to early death or non-response was considered as event at time zero. The Kaplan-Meier method was used to estimate survival rates, differences were compared with the 2-sided log-rank test(5). Cumulative incidence functions for competing events were estimated according to Kalbfleisch and Prentice(6) and compared with Gray's test(7). The Cox regression model was used to estimate hazard ratios and their 95% confidence interval for prognostic factors(8). Statistical analyses were conducted using SAS (SAS-PC, Version 9.1, Cary, NC: SAS Institute Inc.) or SPSS (IBM Deutschland GmbH,

Ehningen, Germany). The level for claiming statistical significance was set at P <0.05.

Supplementary Table 1. Treatment details of protocol AIEOP-BFM ALL 2000.

**Treatment phase/drug**[a]	**Single or daily dose**	**Days of application per phase**[a]
<u>Prephase</u>		
Prednisone (PO/IV)	60 mg/m^2/d	1-7
Methotrexate (IT)	12 mg/dose[b]	1
<u>Induction /</u>		
Protocol IA		
Prednisone/Prednisolone (PO/IV) *or*[c]	60 mg/m^2/d	8-28[d]
Dexamethasone (PO/IV)	10 mg/m^2/d	8-28[d]
Vincristine (IV)	1.5 mg/m^2/dose (max 2 mg)	8, 15, 22, 29
Daunorubicin (PI over 1 h)	30 mg/m^2/dose	8, 15, 22, 29
L-Asparaginase (PI over 1 h)	5000 IU/m^2/dose	12, 15, 18, 21, 24, 27, 30, 33
Methotrexate (IT)	12 mg/dose[b]	12, 33[e]
<u>Consolidation</u>		
Protocol IB		
Cyclophosphamide (PI over 1 h)	1000 mg/m^2/dose	36, 64
Cytarabine (IV)	75 mg/m^2/dose	38-41, 45-48, 52-55, 59-62
6-Mercaptopurine (PO)	60 mg/m^2/d	36-63
Methotrexate (IT)	12 mg/dose[b]	45, 59
<u>Extra-Compartment Therapy (only SR/IR)</u>		
Protocol M		
6-Mercaptopurine (PO)	25 mg/m^2/d	1-56
Methotrexate (PI over 24 h)[f]	5000 mg/m^2/dose	8, 22, 36, 50
Methotrexate (IT)	12 mg/dose[b]	8, 22, 36, 50
<u>Intensive Consolidation (only HR)</u>		
Element HR-1'	20 mg/m^2/d	1-5
Dexamethasone (PO/IV)	1.5 mg/m^2 (max 2 mg)	1, 6
Vincristine (IV)	5000 mg/m^2/dose	1
Methotrexate (PI over 24 h)[f]	200 mg/m^2/dose	2-4 (5 doses, 12 h intervals)
Cyclophosphamide (PI over 1 h)	2 g/m^2/dose	5 (2 doses, 12 h interval)
Cytarabine (PI over 3 h)	25,000 IU/m^2/dose	6, 11
L-Asparaginase (PI over 2 h)	12/30/10 mg/dose[b]	1
Methotrexate/Cytarabine/ Prednisolone (IT)		
Element HR-2'		
Dexamethasone (PO/IV)	20 mg/m^2/d	1-5
Vindesine (IV)	3 mg/m^2/dose (max 5 mg)	1, 6
Methotrexate (PI over 24 h)[f]	5000 mg/m^2/dose	1
Ifosfamide (PI over 1 h)	800 mg/m^2/dose	2-4 (5 doses, 12 h intervals)
Daunorubicin (PI over 24 h)	30 mg/m^2/dose	5
L-Asparaginase (PI over 2 h)	25,000 IU/m^2/dose	6, 11
Methotrexate/Cytarabine/ Prednisolone (IT)	12/30/10 mg/dose[b]	1[g]

Treatment phase/drug[a]	Single or daily dose	Days of application per phase[a]
Element HR-3'		
Dexamethasone (PO/IV)	20 mg/m^2/d	1-5
Cytarabine (PI over 3 h)	2 g/m^2/dose	1-2 (4 doses, 12 h intervals)
Etoposide (PI over 1 h)	100 mg/m^2/dose	3-5 (5 doses, 12 h intervals)
L-Asparaginase (PI over 2 h)	25,000 IU/m^2/dose	6, 11
Methotrexate/Cytarabine/ Prednisolone (IT)	12/30/10 mg/dose[b]	5
Reinduction		
Protocol II		
Dexamethasone (PO/IV)	10 mg/m^2/d	1-21[d]
Vincristine (IV)	1.5 mg/m^2/dose (max 2 mg)	8, 15, 22, 29
Doxorubicin (PI over 1 h)	30 mg/m^2/dose	8, 15, 22, 29
L-Asparaginase (PI over 1 h)	10,000 IU/m^2/dose	8, 11, 15, 18
Cyclophosphamide (PI over 1 h)	1000 mg/m^2/dose	36
Cytarabine (IV)	75 mg/m^2/dose	38-41, 45-48
6-Thioguanine (PO)	60 mg/m^2/d	36-49
Methotrexate (IT)	12 mg/dose[b]	45, 59[g]
Protocol III		
Dexamethasone (PO)	10 mg/m^2/d	1-14[d]
Vincristine (IV)	1.5 mg/m^2/dose (max 2 mg)	1, 8
Doxorubicin (PI over 1 h)	30 mg/m^2/dose	1, 8
L-Asparaginase (PI over 1 h)	10,000 IU/m^2/dose	1, 4, 8, 11
Cyclophosphamide (PI over 1 h)	500 mg/m^2/dose	15
Cytarabine (IV)	75 mg/m^2/dose	17-20, 24-27
6-Thioguanine (PO)	60 mg/m^2/d	15-28
Methotrexate (IT)	12 mg/dose[b]	17, 24[g]
Interim Maintenance		
Methotrexate (PO)	20 mg/m^2/dose[h]	once a week
6-Mercaptopurine (PO)	50 mg/m^2/d[i]	daily
Maintenance[i]		
Methotrexate (PO)	20 mg/m^2/dose[h]	once a week
6-Mercaptopurine (PO)	50 mg/m^2/d[i]	daily
Cranial irradiadion	12 Gy/18 Gy/24 Gy	

[a] PO indicates orally; IV, intravenous push; PI, intravenous infusion; IT, intrathecally; adjustments of time schedule were allowed if clinical condition and bone marrow recovery were inadequate

[b] Doses of IT drugs were adjusted for children <3 years of age

[c] Randomization

[d] Steroids were tapered over 9 additional days

[e] Additional IT therapy on day 18 and 27 was administered to patients with CNS status CNS3 and CNS2 or TLP+

[f] A loading dose of 10% was infused over 30 min, the remaining 90% over 23.5 h. Leucovorin rescue was given at hour 42, 48, and 54 (each 15 mg/m2). Doses of leucovorin rescue were adjusted, if MTX levels were >1.0 µmol/L at hour 42 or later. If the MTX level at hour 54 was >0.25 µmol/L, rescue was continued at six-hour intervals until MTX levels were ≤ 0.25 µmol/L.

[g] Patients with CNS status CNS 3 received additional IT therapy on day 5 in element HR-2', on day 1 and 18 in Protocol II and on day 1 in Protocol III

[h] Doses were adjusted to white blood cell count (WBC, target range 2.0-3.0 x10^9/L)

[i] Maintenance was given from the end of intensive chemotherapy until 104 weeks after diagnosis

Supplementary Table 2. Clinical characteristics of the patients in the study cohort by severity of bilirubin toxicity during induction/consolidation (protocols IA/IB, n=1547).

		**CTC grade 0** n=540(n%)	**CTC grades 1-2** n=825(n%)	**CTC grades 3-4** n=182(n%)	***P***[a]
Sex	Male	300 (56%)	464 (56%)	97 (53%)	
	Female	240 (44%)	361 (44%)	85 (47%)	0.768
Age at diagnosis of ALL [y]	<6	372 (69%)	430 (52%)	53 (29%)	
	≥6 <10	93 (17%)	172 (21%)	33 (18%)	
	≥10	75 (14%)	223 (27%)	96 (53%)	<0.001
Immunophenotype	B-cell ALL	461 (85%)	683 (83%)	150 (82%)	
	T-cell ALL	55 (10%)	117 (14%)	29 (16%)	0.062
	Other/not characterized[b]	24 (4%)	25 (3%)	3 (2%)	
White blood cell count at diagnosis of ALL [/µL]	<10000	261 (48%)	403 (49%)	85 (47%)	
	≥10000 <50000	187 (35%)	265 (32%)	58 (32%)	
	≥50000 <100000	52 (10%)	80 (10%)	15 (8%)	
	≥100000	40 (7%)	76 (9%)	24 (13%)	0.395
	Unknown	0 (0%)	1 (0%)	0 (0%)	
CNS positivity[c]	No	508 (94%)	767 (93%)	165 (91%)	
	Yes	13 (2%)	21 (3%)	12 (7%)	0.010
	Unknown	19 (4%)	37 (4%)	5 (3%)	
Hyperdiploidy[d]	No	303 (56%)	507 (61%)	120 (66%)	
	Yes	105 (19%)	119 (14%)	19 (10%)	0.003
	Unknown	132 (24%)	199 (24%)	43 (24%)	
ETV6-RUNX1 rearrangement	Negative	380 (70%)	558 (68%)	130 (71%)	
	Positive	120 (22%)	196 (24%)	35 (19%)	0.389
	Unknown	40 (7%)	71 (9%)	17 (9%)	
Prednisone response[e]	Good	492 (91%)	738 (89%)	160 (88%)	
	Poor	40 (7%)	78 (9%)	18 (10%)	0.366
	Unknown	8 (1%)	9 (1%)	4 (2%)	
MRD risk group[f]	Standard	223 (41%)	355 (43%)	73 (40%)	
	Intermediate	251 (46%)	360 (44%)	72 (40%)	
	High	32 (6%)	55 (7%)	17 (9%)	0.389
	Unknown	34 (6%)	55 (7%)	20 (11%)	
Final risk group[g]	Standard	167 (31%)	267 (32%)	51 (28%)	
	Intermediate	303 (56%)	432 (52%)	100 (55%)	
	High	69 (13%)	125 (15%)	31 (17%)	0.410
	Other/Unknown	1 (0%)	1 (0%)	0 (0%)	
Maximum transaminase levels during protocol IA/IB[h]	CTC grade 0	71 (13%)	23 (3%)	2 (1%)	
	CTC grades 1-2	249 (46%)	376 (46%)	53 (29%)	
	CTC grades 3-4	212 (39%)	426 (52%)	127 (70%)	<0.001
	Unknown	8 (1%)	0 (0%)	0 (0%)	
Maximum bilirubin levels during protocol IA[i]	CTC grade 0	499 (92%)	115 (14%)	6 (3%)	
	CTC grades 1-2	0 (0%)	690 (84%)	17 (9%)	
	CTC grades 3-4	0 (0%)	0 (0%)	158 (87%)	<0.001
	Unknown	41 (8%)	20 (2%)	1 (1%)	
Maximum bilirubin levels during protocol IB[i]	CTC grade 0	501 (93%)	307 (37%)	35 (19%)	
	CTC grades 1-2	0 (0%)	490 (59%)	85 (47%)	
	CTC grades 3-4	0 (0%)	0 (0%)	50 (27%)	<0.001
	Unknown	39 (7%)	28 (3%)	12 (7%)	
Maximum bilirubin levels during the entire course of therapy[j]	CTC grade 0	412 (76%)	0 (0%)	0 (0%)	
	CTC grades 1-2	123 (23%)	767 (93%)	0 (0%)	
	CTC grades 3-4	5 (1%)	58 (7%)	182 (100%)	<0.001

Abbreviations: CNS: central nervous system; CTC: Common Toxicity Criteria of the National Cancer Institute version 2; UNL: Upper normal limit.

[a] *P*-values resulting from X^2 tests: Patients of the study cohort with moderate (CTC grades 1-2) and high (CTC grades 3-4) hyperbilirubinemia, during induction and/or consolidation (protocols IA/IB) of the AIEOP-BFM ALL protocol versus patients with normal levels (CTC grade 0, ≤17.1 µmol/L(UNL)).

[b] One patient was diagnosed with acute undifferentiated leukemia and no immunophenotype information was available for fifty-one patients.

[c] CNS negative, puncture nontraumatic without leukemic blasts in the cerebrospinal fluid (CSF) after cytocentrifugation; CNS positive, puncture nontraumatic with >5 leukocytes/µL in the CSF with identifiable blasts.

[d] Defined by cytogenetics (>50 chromosomes) or by flow cytometric analyses of the ratio of DNA content of leukemic G0/G1 cells to normal diploid lymphocytes (≥1.16).

[e] Good <1000 leukemic blasts/µL peripheral blood on treatment day 8; poor ≥1000 blasts/µL.

[f] Risk stratification based on minimal residual disease (MRD) analysis for ERG: Standard risk, MRD-negative on treatment day33 and 78; high risk, leukemic cell load $\geq 5\times10^{-4}$ on treatment day 78; all other results correspond to intermediate risk.

[g] Treatment group according to risk stratification including all relevant diagnostic parameters.

[h] Toxicity grading of the alanine and aspartate transaminase serum activity levels during induction/consolidation (protocols IA/IB) was according to CTC, considering 20 U/L as the UNL.

[i] Bilirubin toxicity grading during induction/consolidation (protocols IA/IB) was according to the CTC, with grade 0 corresponding to total serum levels ≤UNL, grade 1 to levels >UNL to 1.5xUNL, grade 2 levels >1.5x UNL to 3.0x UNL, grade 3 levels >3.0x UNL to 10.0x UNL and grade 4 to levels >10.0x UNL.

[j] The highest individual bilirubin toxicity level throughout the entire treatment course under investigation. Toxicity grading was as above (CTC).

Supplementary Table 3. Characteristics of the patients in the GWAS discovery cohort by serum bilirubin levels during induction/consolidation (n=650).

		Patients without hyperbilirubinemia (n=215)	(%)	Patients with hyperbilirubinemia (n=435)	(%)	P^a
Sex	Male	120	(56%)	253	(58%)	
	Female	95	(44%)	182	(42%)	0.569
Age at diagnosis of ALL [years]	<6	143	(67%)	192	(44%)	
	≥6 <10	35	(16%)	89	(20%)	
	≥10	37	(17%)	154	(35%)	<0.001
Immunophenotype	B cell ALL	182	(85%)	330	(76%)	
	T cell ALL	32	(15%)	101	(23%)	0.012
	Other/not characterized[b]	1	(0%)	4	(1%)	
White blood cell count at diagnosis of ALL [/µL]	<10000	83	(39%)	179	(41%)	
	≥10000 <50000	83	(39%)	142	(33%)	
	≥50000 <100000	24	(11%)	50	(11%)	
	≥100000	25	(12%)	64	(15%)	0.437
CNS positivity[c]	No	201	(93%)	395	(91%)	
	Yes	7	(3%)	18	(4%)	0.552
	Unknown	7	(3%)	22	(5%)	
Hyperdiploidy[d]	No	122	(57%)	254	(58%)	
	Yes	44	(20%)	65	(15%)	0.125
	Unknown	49	(23%)	116	(27%)	
ETV6-RUNX1 rearrangement	Negative	191	(89%)	370	(85%)	
	Positive	4	(2%)	18	(4%)	0.122
	Unknown	20	(9%)	47	(11%)	
Prednisone response[e]	Good	194	(90%)	373	(86%)	
	Poor	21	(10%)	56	(13%)	0.225
	Unknown	0	(0%)	6	(1%)	
MRD risk group[f]	Standard	74	(34%)	167	(38%)	
	Intermediate	117	(54%)	213	(49%)	
	High	17	(8%)	38	(9%)	0.458
	Unknown	7	(3%)	17	(4%)	
Final risk group[g]	Standard	50	(23%)	125	(29%)	
	Intermediate	127	(59%)	228	(52%)	
	High	38	(18%)	82	(19%)	0.237
Bilirubin levels at diagnosis [µmol/L][h]	≤17.1	114	(53%)	233	(54%)	
	>17.1	2	(1%)	19	(4%)	0.025
	Unknown	99	(46%)	183	(42%)	
Maximum transaminase levels during protocols IA/IB[i]	CTC grade 0	29	(13%)	15	(3%)	
	CTC grades 1-2	96	(45%)	193	(44%)	
	CTC grades 3-4	88	(41%)	227	(52%)	<0.001
	Unknown	2	(1%)	0	(0%)	
Maximum bilirubin levels during protocol IA[j]	CTC grade 0	199	(93%)	55	(13%)	
	CTC grades 1-2	0	(0%)	313	(72%)	
	CTC grades 3-4	0	(0%)	59	(14%)	<0.001
	Unknown	16	(7%)	8	(2%)	
Maximum bilirubin levels during protocol IB[j]	CTC grade 0	199	(93%)	153	(35%	
	CTC grades 1-2	0	(0%)	248	(57%)	
	CTC grades 3-4	0	(0%)	21	(5%)	<0.001
	Unknown	16	(7%)	13	(3%)	
Maximum bilirubin levels during the entire course of therapy[k]	CTC grade 0	166	(77%)	0	(0%)	
	CTC grades 1-2	47	(22%)	346	(80%)	
	CTC grades 3-4	2	(1%)	89	(20%)	<0.001

Abbreviations: CNS: central nervous system; CTC: Common Toxicity Criteria of the National Cancer Institute version 2; GWAS: genome-wide association study.

[a] *P*-values resulting from X^2 or Fisher's exact test: Patients of the discovery cohort with hyperbilirubinemia, i.e. bilirubin levels >17.1 µmol/L during induction and/or consolidation (protocols IA/IB) of the AIEOP-BFM ALL protocol (CTC grades 1-4, GWAS cases) versus patients with normal levels ≤17.1 µmol/L (CTC grade 0, GWAS controls).

[b] One patient was diagnosed with acute undifferentiated leukemia and no immunophenotype information was available for four patients.

[c] CNS negative, puncture nontraumatic without leukemic blasts in the cerebrospinal fluid (CSF) after cytocentrifugation; CNS positive, puncture nontraumatic with >5 leukocytes /µL in the CSF with identifiable blasts.

[d] Defined by cytogenetics (>50 chromosomes) or by flow cytometric analyses of the ratio of DNA content of leukemic G0/G1 cells to normal diploid lymphocytes (≥1.16).

[e] Good <1000 leukemic blasts/µL peripheral blood on treatment day 8; poor ≥1000 blasts/µL.

[f] Risk stratification based on minimal residual disease (MRD) analysis for ERG: Standard risk, MRD-negative on treatment day33 and 78; high risk, leukemic cell load $\geq 5 \times 10^{-4}$ on treatment day 78; all other results correspond to intermediate risk.

[g] Treatment group according to risk stratification including all relevant diagnostic parameters.

[h] Total serum bilirubin levels at day 0 of the therapy; levels ≤17.1 µmol/L corresponding to the upper normal level (UNL) and CTC grade 0.

[i] Toxicity grading of the alanine and aspartate transaminase serum activity levels during induction/consolidation (protocols IA/IB) was according to CTC, considering 20 U/L as the UNL.

[j] Bilirubin toxicity grading during induction/consolidation (protocols IA/IB) was according to the CTC, with grade 0 corresponding to total serum levels ≤UNL, grade 1 to levels >UNL to 1.5xUNL, grade 2 levels >1.5x UNL to 3.0x UNL, grade 3 levels >3.0x UNL to 10.0x UNL and grade 4 to levels >10.0x UNL.

[k] The highest individual bilirubin toxicity level throughout the entire treatment course under investigation (Suppl. Figure 3). Toxicity grading was as above (CTC).

Supplementary Table 4. Characteristics of the patients in the GWAS discovery cohort compared to all patients of the study cohort with toxicity information.

		**Patients not included in the discovery GWAS** (n=897)	(%)	**Patients included in the discovery GWAS** (n=650)	(%)	***P***[a]
Sex	Male	488	(54%)	373	(57%)	
	Female	409	(46%)	277	(43%)	0.244
Age at diagnosis of ALL [y]	<6	520	(58%)	335	(52%)	
	≥6 <10	174	(19%)	124	(19%)	
	≥10	203	(23%)	191	(29%)	0.008
Immunophenotype	B cell ALL	782	(87%)	512	(79%)	
	T cell ALL	68	(8%)	133	(20%)	<0.001
	Other/not characterized[b]	47	(5%)	5	(1%)	
White blood cell count at diagnosis of ALL[/μL]	<10000	487	(54%)	262	(40%)	
	≥10000 <50000	285	(32%)	225	(35%)	
	≥50000 <100000	73	(8%)	74	(11%)	
	≥100000	51	(6%)	89	(14%)	<0.001
	Unknown	1	(0%)	0	(0%)	
CNS positivity[c]	No	844	(94%)	596	(92%)	
	Yes	21	(2%)	25	(4%)	0.079
	Unknown	32	(4%)	29	(4%)	
Hyperdiploidy[d]	No	554	(62%)	376	(58%)	
	Yes	134	(15%)	109	(17%)	0.212
	Unknown	209	(23%)	165	(25%)	
ETV6-RUNX1 rearrangement	Negative	507	(57%)	561	(86%)	
	Positive	329	(37%)	22	(3%)	<0.001
	Unknown	61	(7%)	67	(10%)	
Prednisone response[e]	Good	823	(92%)	567	(87%)	
	Poor	59	(7%)	77	(12%)	<0.001
	Unknown	15	(2%)	6	(1%)	
MRD risk group[f]	Standard	410	(46%)	241	(37%)	
	Intermediate	353	(39%)	330	(51%)	
	High	49	(5%)	55	(8%)	<0.001
	Unknown	85	(9%)	24	(4%)	
Final risk group[g]	Standard	310	(35%)	175	(27%)	
	Intermediate	480	(54%)	355	(55%)	
	High	105	(12%)	120	(18%)	<0.001
	Other/Unknown	2	(0%)	0	(0%)	
Maximum transaminase levels during protocols IA/IB[h]	CTC grade 0	52	(6%)	44	(7%)	
	CTC grades 1-2	389	(43%)	289	(44%)	
	CTC grades 3-4	450	(50%)	315	(48%)	0.640
	Unknown	6	(1%)	2	(0%)	
Maximum bilirubin levels during protocol IA/IB[i]	CTC grade 0	325	(36%)	215	(33%)	
	CTC grades 1-2	458	(51%)	367	(56%)	
	CTC grades 3-4	114	(13%)	68	(10%)	0.093
Maximum bilirubin levels during protocol IA[i]	CTC grade 0	366	(41%)	254	(39%)	
	CTC grades 1-2	394	(44%)	313	(48%)	
	CTC grades 3-4	99	(11%)	59	(9%)	0.206
	Unknown	38	(4%)	24	(4%)	
Maximum bilirubin levels during protocol IB[i]	CTC grade 0	491	(55%)	352	(54%)	
	CTC grades 1-2	327	(36%)	248	(38%)	
	CTC grades 3-4	29	(3%)	21	(3%)	0.875
	Unknown	50	(6%)	29	(4%)	
Maximum bilirubin during entire therapy[j]	CTC grade 0	246	(27%)	166	(26%)	
	CTC grades 1-2	497	(55%)	393	(60%)	
	CTC grades 3-4	154	(17%)	91	(14%)	0.102

Abbreviations: CNS: central nervous system; CTC: Common Toxicity Criteria of the National Cancer Institute version 2; GWAS: genome-wide association study.

[a] *P*-values resulting from X^2 or Fisher's exact test: Patients of the GWAS discovery cohort were genotyped on Human Omni1-Quad v1 arrays (Illumina, San Diego, CA, USA) as previously described(9) and were compared here to patients from the study cohort who were not included in previous genotyping but have available toxicity information. Due to prior selection, our discovery cohort included a higher number of older patients, more T cell ALL patients, more prednisone poor responders, more patients with high and intermediate MRD risk and less patients with the *ETV6-RUNX1* rearrangement than the overall study population.

[b] One patient was diagnosed with acute undifferentiated leukemia and no immunophenotype information was available for fifty-one patients.

[c] CNS negative, puncture nontraumatic without leukemic blasts in the cerebrospinal fluid (CSF) after cytocentrifugation; CNS positive, puncture nontraumatic with >5 leukocytes /µL in the CSF with identifiable blasts.

[d] Defined by cytogenetics (>50 chromosomes) or by flow cytometric analyses of the ratio of DNA content of leukemic G0/G1 cells to normal diploid lymphocytes (≥1.16).

[e] Good <1000 leukemic blasts/µL peripheral blood on treatment day 8; poor ≥1000 blasts/µL.

[f] Risk stratification based on minimal residual disease (MRD) analysis for ERG: Standard risk, MRD-negative on treatment day33 and 78; high risk, leukemic cell load $\geq 5 \times 10^{-4}$ on treatment day 78; all other results correspond to intermediate risk.

[g] Treatment group according to risk stratification including all relevant diagnostic parameters.

[h] Toxicity grading of the alanine and aspartate transaminase serum activity levels during induction/consolidation (protocols IA/IB) was according to CTC, considering 20 U/L as the UNL.

[i] Bilirubin toxicity grading during induction/consolidation (protocols IA/IB) was according to the CTC, with grade 0 corresponding to total serum levels ≤UNL, grade 1 to levels >UNL to 1.5xUNL, grade 2 levels >1.5x UNL to 3.0x UNL, grade 3 levels >3.0x UNL to 10.0x UNL and grade 4 to levels >10.0x UNL.

[j] The highest individual bilirubin toxicity level throughout the entire treatment course under investigation (Suppl. Figure 3). Toxicity grading was as above (CTC).

Supplementary Table 5. Summary of genome-wide association analysis for therapy-related hyperbilirubinemia during induction/consolidation (protocols IA/IB).

Variant Identifier	X^2 allelic association analysis		Allelic association without covariate adjustment		Allelic association including covariates		Allele information			SNV information according to reference data available					
**SNV**	***P*[a]**	**OR**	***P*[b]**	**OR (95% CI)[b]**	***P*[c]**	**OR (95% CI)[c]**	**Minor allele (A1)[d]**	**MAF (A1, CAS)[e]**	**MAF (A1, CTR)[e]**	**Gene symbol**	**CHR[f]**	**BP[f]**	**MAF (global)[g]**	**MAF (EUR)[g]**	**HGVS expression[f]**
rs6744284	7.3x10^{-8}	2.1	1.8x10^{-7}	2.1(1.6-2.7)	1.2x10^{-7}	2.1(1.6-2.8)	T	0.369	0.221	*UGT1A4*	2	234625297	T=0.390	T=0.298	NC_000002.11:g.234625297C>T
rs3771341	2.4x10^{-7}	2.0	5.3x10^{-7}	2.0(1.5-2.6)	2.1x10^{-7}	2.1(1.6-2.8)	A	0.360	0.219	*UGT1A1*	2	234673239	A=0.330	A=0.308	NC_000002.11:g.234673239G>A
rs17862875	4.7x10^{-7}	2.0	9.8x10^{-7}	2.0(1.5-2.6)	3.5x10^{-7}	2.1(1.6-2.8)	A	0.359	0.221	*DNAJB3*	2	234649302	A=0.295	A=0.274	NC_000002.11:g.234649302G>A
rs887829	5.2x10^{-7}	1.9	7.3x10^{-7}	1.9(1.5-2.5)	3.4x10^{-7}	2.0(1.6-2.7)	A	0.402	0.261	*UGT1A1*	2	234668570	T=0.354	T=0.298	NC_000002.11:g.234668570C>T
rs17863787	6.6x10^{-7}	1.9	1.2x10^{-6}	1.9(1.5-2.5)	1.1x10^{-6}	2.0(1.5-2.6)	G	0.392	0.252	*UGT1A6*	2	234611094	G=0.263	G=0.285	NC_000002.11:g.234611094T>G
rs1105879	7.1x10^{-7}	1.9	1.3x10^{-6}	1.9(1.5-2.4)	1.5x10^{-6}	1.9(1.5-2.5)	C	0.427	0.284	*UGT1A6*	2	234602202	C=0.325	C=0.333	NC_000002.11:g.234602202A>C
rs6742078	7.4x10^{-7}	1.9	9.7x10^{-7}	1.9(1.5-2.5)	5.2x10^{-7}	2.0(1.5-2.7)	T	0.400	0.261	*UGT1A1*	2	234672639	T=0.348	T=0.298	NC_000002.11:g.234672639G>T
rs4148324	7.4x10^{-7}	1.9	9.7x10^{-7}	1.9(1.5-2.5)	5.2x10^{-7}	2.0(1.5-2.7)	G	0.400	0.261	*UGT1A1*	2	234672722	G=0.353	G=0.298	NC_000002.11:g.234672722T>G
rs4148325	7.4x10^{-7}	1.9	9.7x10^{-7}	1.9(1.5-2.5)	5.2x10^{-7}	2.0(1.5-2.7)	T	0.400	0.261	*UGT1A1*	2	234673309	T=0.354	T=0.298	NC_000002.11:g.234673309C>T
rs1105880	9.3x10^{-7}	1.9	1.5x10^{-6}	1.9(1.4-2.4)	1.6x10^{-6}	1.9(1.5-2.5)	G	0.426	0.286	*UGT1A6*	2	234601965	G=0.343	G=0.333	NC_000002.11:g.234601965A>G
rs929596	1.2x10^{-6}	2.0	3.1x10^{-6}	1.9(1.5-2.5)	7.6x10^{-7}	2.1(1.6-2.8)	G	0.332	0.202	*UGT1A1*	2	234674476	G=0.324	G=0.254	NC_000002.11:g.234674476A>G
rs10065220	5.1x10^{-6}	0.6	7.2x10^{-6}	0.6(0.4-0.7)	2.3x10^{-5}	0.6(0.4-0.7)	A	0.305	0.433	*FBXL7*	5	15384680	A=0.251	A=0.370	NC_000005.9:g.15384680G>A
rs2076549	1.0x10^{-5}	0.6	3.5x10^{-5}	0.6(0.5-0.8)	7.3x10^{-5}	0.6(0.5-0.8)	G	0.298	0.421	*SULF2*	20	46290316	G=0.413	G=0.325	NC_000020.10:g.46290316A>G
rs2070959	1.0x10^{-5}	1.8	1.5x10^{-5}	1.8(1.4-2.3)	1.1x10^{-5}	1.8(1.4-2.4)	G	0.397	0.272	*UGT1A6*	2	234602191	G=0.278	G=0.310	NC_000002.11:g.234602191A>G
rs13009407	1.1x10^{-5}	1.9	2.3x10^{-5}	1.8(1.4-2.4)	5.7x10^{-6}	2.0(1.5-2.6)	G	0.311	0.195	*DNAJB3*	2	234652347	G=0.146	G=0.236	NC_000002.11:g.234652347C>G
rs7592624	1.2x10^{-5}	0.6	1.3x10^{-5}	0.6(0.5-0.7)	2.1x10^{-5}	0.6(0.5-0.7)	G	0.379	0.507	*UGT1A6*	2	234602906	G=0.444	G=0.447	NC_000002.11:g.234602906G>A
rs1439494	1.2x10^{-5}	0.5	2.2x10^{-5}	0.5(0.4-0.7)	2.8x10^{-5}	0.5(0.4-0.7)	A	0.137	0.235	*MIR924HG*	18	36341319	A=0.113	A=0.192	NC_000018.9:g.36341319G>A
rs10168416	1.2x10^{-5}	1.8	1.8x10^{-5}	1.7(1.4-2.3)	1.4x10^{-5}	1.8(1.4-2.3)	G	0.395	0.272	*UGT1A6*	2	234597087	G=0.276	G=0.310	NC_000002.11:g.234597087C>G
rs2741045	1.3x10^{-5}	1.8	1.8x10^{-5}	1.8(1.4-2.3)	1.1x10^{-5}	1.9(1.4-2.5)	T	0.352	0.233	*UGT1A9*	2	234580140	T=0.159	T=0.274	NC_000002.11:g.234580140C>T
rs1821763	1.4x10^{-5}	1.9	1.0x10^{-5}	1.9(1.4-2.6)	1.1x10^{-5}	2.0(1.5-2.7)	C	0.301	0.188	*KCTD3/ USH2*	1	216165271	G=0.227	G=0.295	NC_000001.10:g.216165271G>A

[a] Asymptotic *P*-values resulting from allelic X^2 test statistic (plink).

[b] Asymptotic *P*-values, odds ratios (OR) and 95% confidence intervals (CI) resulting from unadjusted logistic regression analysis.

[c] Age at diagnosis of acute lymphoblastic leukemia and immunophenotype were included as covariates in multivariate association testing (logistic regression), resulting asymptotic *P*-values for t-statistic, odds ratios (OR) and 95% confidence intervals (CI) are given for each of the identified twenty most associated variants.
[d] Minor allele is reported according to Illumina genotyping chip information.
[e] Minor allele frequencies (MAF) detected in genotyped cases (CAS) and controls (CTR) included in the current genome-wide association study.
[f] Chromosome (CHR), base pair position (BP) and Human Genome Variation Society (HGVS) expressions are according to GRCh37.p13 (hg19).
[g] 1000 Genomes Project, phase3; Minor allele is reported in forward orientation.

Supplementary Table 6. Allelic association of hyperbilirubinemia phenotype with the 20 most strongly associated variants around rs6744284 after genotype imputation.

Variant identifier	Typed or imputed	r^2(LD)[a]	Minor allele[b]	Minor allele frequency[c]		Allelic association		
				Controls (CTC 0)	Cases (CTC 1-4)	X^2	OR (95%CI)	P^d
rs6715829	imputed	0.695	T	0.414	0.261	29.3	2.00(1.55-2.58)	$6.3x10^{-8}$
rs6744284	typed	1	T	0.369	0.221	29.0	2.06(1.58-2.69)	$7.3x10^{-8}$
rs6747843	imputed	0.883	A	0.362	0.219	27.5	2.03(1.55-2.65)	$1.6x10^{-7}$
rs6714634	imputed	0.883	C	0.362	0.219	27.5	2.03(1.55-2.65)	$1.6x10^{-7}$
rs10929302	imputed	0.883	A	0.362	0.219	27.5	2.03(1.55-2.65)	$1.6x10^{-7}$
rs9711503	imputed	0.883	C	0.362	0.219	27.5	2.03(1.55-2.65)	$1.6x10^{-7}$
rs2885296	imputed	0.88	C	0.361	0.219	27.1	2.02(1.55-2.64)	$2.0x10^{-7}$
rs111741722	imputed	0.749	G	0.402	0.256	27.0	1.96(1.52-2.53)	$2.0x10^{-7}$
rs11695484	imputed	0.883	G	0.360	0.219	26.7	2.01(1.54-2.62)	$2.4x10^{-7}$
rs17864701	imputed	0.889	T	0.358	0.219	25.9	1.99(1.52-2.6)	$3.6x10^{-7}$
rs3806592	imputed	0.936	T	0.354	0.216	25.6	1.99(1.52-2.6)	$4.2x10^{-7}$
rs112132688	imputed	0.899	A	0.364	0.226	25.5	1.97(1.51-2.57)	$4.3x10^{-7}$
rs7567229	imputed	0.774	A	0.395	0.254	25.5	1.93(1.49-2.49)	$4.4x10^{-7}$
rs17862875	typed	0.896	A	0.359	0.221	25.4	1.97(1.51-2.57)	$4.7x10^{-7}$
rs34352510	imputed	0.896	C	0.359	0.221	25.4	1.97(1.51-2.57)	$4.7x10^{-7}$
rs9711502	imputed	0.749	C	0.402	0.261	25.3	1.91(1.48-2.47)	$5.0x10^{-7}$
rs13401281	imputed	0.896	G	0.364	0.228	24.7	1.94(1.49-2.53)	$6.8x10^{-7}$
rs138869941	imputed	0.896	T	0.364	0.228	24.7	1.94(1.49-2.53)	$6.8x10^{-7}$
rs6742078	typed	0.742	T	0.400	0.261	24.5	1.89(1.47-2.44)	$7.4x10^{-7}$
rs4148324	typed	0.742	G	0.400	0.261	24.5	1.89(1.47-2.44)	$7.4x10^{-7}$

[a] Magnitude of linkage disequilibrium (LD) with the lead variant given as r^2.
[b] Minor allele according to genotyping data.
[c] Minor allele frequencies as detected for the derivative cohort, including 435 cases with hyperbilirubinemia (CTC grades 1-4) and 215 controls with normal bilirubin levels during induction/consolidation (protocols IA/IB). Toxicity grading was according to the Common Toxicity Criteria of the National Cancer Institute version 2 (CTC).
[d] Asymptotic p-value for allelic test X^2 statistic.

Supplementary Table 7. Genotypic association between hyperbilirubinemia phenotype and the 20 most strongly associated SNV around rs6744284 after genotype imputation.

Variant identifier	Genotype counts in unadjusted analysis [a, b]		Genotypic association without covariate adjustment(n=650)[b]				Genotype counts in adjusted analysis [a, c]		Genotypic association adjusted for age and immunophenotype(n=645)[c]			
	**Controls**	**Cases**	**OR_{het}(95%CI)**	***P***	**OR_{hom}(95%CI)**	***P***	**Controls**	**Cases**	**OR_{het}(95%CI)**	***P***	**OR_{hom}(95%CI)**	***P***
rs6715829	13/86/116	75/210/150	1.89(1.33-2.68)	0.0004	4.46(2.36-8.43)	<0.0001	13/85/116	75/207/149	1.90(1.33-2.73)	0.0005	4.51(2.36-8.63)	<0.0001
rs6744284	7/81/127	63/195/177	1.73(1.22-2.44)	0.0019	6.46(2.86-14.57)	<0.0001	7/80/127	63/192/176	1.85(1.29-2.65)	0.0007	6.50(2.85-14.82)	<0.0001
rs6747843	6/82/127	62/191/182	1.63(1.15-2.29)	0.0056	7.21(3.03-17.18)	<0.0001	6/81/127	62/189/180	1.80(1.26-2.58)	0.0012	7.38(3.07-17.79)	<0.0001
rs6714634	6/82/127	62/191/182	1.63(1.15-2.29)	0.0056	7.21(3.03-17.18)	<0.0001	6/81/127	62/189/180	1.80(1.26-2.58)	0.0012	7.38(3.07-17.79)	<0.0001
rs10929302	6/82/127	62/191/182	1.63(1.15-2.29)	0.0056	7.21(3.03-17.18)	<0.0001	6/81/127	62/189/180	1.80(1.26-2.58)	0.0012	7.38(3.07-17.79)	<0.0001
rs9711503	6/82/127	62/191/182	1.63(1.15-2.29)	0.0056	7.21(3.03-17.18)	<0.0001	6/81/127	62/189/180	1.80(1.26-2.58)	0.0012	7.38(3.07-17.79)	<0.0001
rs2885296	6/82/127	62/190/183	1.61(1.14-2.27)	0.0068	7.17(3.01-17.08)	<0.0001	6/81/127	62/188/181	1.79(1.25-2.56)	0.0014	7.36(3.05-17.72)	<0.0001
rs111741722	10/90/115	71/208/156	1.7(1.21-2.41)	0.0025	5.23(2.59-10.59)	<0.0001	10/89/115	71/205/155	1.85(1.29-2.65)	0.0008	5.41(2.64-11.08)	<0.0001
rs11695484	6/82/127	61/191/183	1.62(1.15-2.28)	0.0062	7.06(2.96-16.82)	<0.0001	6/81/127	61/189/181	1.80(1.26-2.58)	0.0012	7.17(2.98-17.28)	<0.0001
rs17864701	6/82/127	61/189/185	1.58(1.12-2.23)	0.0089	6.98(2.93-16.63)	<0.0001	6/81/127	61/187/183	1.76(1.23-2.52)	0.0019	7.08(2.94-17.07)	<0.0001
rs3806592	6/81/128	59/190/186	1.61(1.14-2.28)	0.0064	6.77(2.84-16.14)	<0.0001	6/80/128	59/187/185	1.77(1.24-2.52)	0.0018	6.81(2.82-16.42)	<0.0001
rs112132688	7/83/125	63/191/181	1.59(1.13-2.24)	0.0083	6.22(2.76-14.02)	<0.0001	7/82/125	63/188/180	1.76(1.23-2.52)	0.0019	6.23(2.73-14.21)	<0.0001
rs7567229	12/85/118	69/206/160	1.79(1.26-2.53)	0.001	4.24(2.2-8.19)	<0.0001	12/84/118	69/203/159	1.91(1.33-2.73)	0.0004	4.32(2.21-8.44)	<0.0001
rs17862875	6/83/126	61/190/184	1.57(1.11-2.21)	0.0103	6.96(2.92-16.6)	<0.0001	6/82/126	61/188/182	1.74(1.22-2.49)	0.0023	7.06(2.93-17.02)	<0.0001
rs34352510	6/83/126	61/190/184	1.57(1.11-2.21)	0.0103	6.96(2.92-16.6)	<0.0001	6/82/126	61/188/182	1.74(1.22-2.49)	0.0023	7.06(2.93-17.02)	<0.0001
rs9711502	10/92/113	71/208/156	1.64(1.16-2.31)	0.005	5.14(2.54-10.41)	<0.0001	10/91/113	71/205/155	1.78(1.24-2.55)	0.0016	5.32(2.6-10.9)	<0.0001
rs13401281	7/84/124	63/191/181	1.56(1.11-2.2)	0.0115	6.17(2.73-13.91)	<0.0001	7/83/124	63/188/180	1.73(1.21-2.48)	0.0025	6.19(2.71-14.12)	<0.0001
rs138869941	7/84/124	63/191/181	1.56(1.11-2.2)	0.0115	6.17(2.73-13.91)	<0.0001	7/83/124	63/188/180	1.73(1.21-2.48)	0.0025	6.19(2.71-14.12)	<0.0001
rs6742078	10/92/113	70/208/157	1.63(1.15-2.3)	0.0056	5.04(2.49-10.2)	<0.0001	10/91/113	70/205/156	1.76(1.23-2.52)	0.0019	5.16(2.52-10.57)	<0.0001
rs4148324	10/92/113	70/208/157	1.63(1.15-2.3)	0.0056	5.04(2.49-10.2)	<0.0001	10/91/113	70/205/156	1.76(1.23-2.52)	0.0019	5.16(2.52-10.57)	<0.0001

[a] Counts of the minor allele homozygotes, heterozygotes and of the major allele homozygotes.

[b] Genotypic association was analyzed using binary logistic regression(plink) for 650 patients(435 cases, 215 controls). Odds ratios(OR) are listed for heterozygous(het) and homozygous(hom) genotype minor allele.

[c] Age and immunophenotype information was available for 645 patients(431 cases, 214 controls). For 5 patients immunophenotype information was not available or ambiguous. OR are given for heterozygous(het) and homozygous(hom) genotype of the minor allele

Supplementary Table 8. Adjusted genotypic association of rs6744284 with hyperbilirubinemia during protocols IA/IB and later therapeutic elements, including age and immunophenotype as covariates.

Therapeutic Element	Amount of analyzed subjects[a]			Frequency of CTR/CAS per TT(%)[b]		Frequency of CTR/CAS per TC(%)[b]		Frequency of CTR/CAS per CC(%)[b]		Genotypic association[c]			
	Controls(%)	Cases(%)	Total	CTR(%)	CAS(%)	CTR(%)	CAS(%)	CTR(%)	CAS(%)	OR_{het}(CI 95%)	*P*	OR_{hom}(CI 95%)	*P*
Protocol IA/IB	214(33%)	431(67%)	645	7(10%)	63(90%)	80(29%)	192(71%)	127(42%)	176(58%)	1.85(1.29-2.64)	<0.001	6.50(2.85-14.82)	<0.001
Protocol M	298(61%)	190(39%)	488	17(34%)	33(66%)	123(60%)	82(40%)	158(68%)	75(32%)	1.41(0.95-2.10)	0.088	3.93(2.04-7.56)	<0.001
Protocol II/III	345(76%)	110(24%)	455	16(37%)	27(63%)	136(74%)	47(26%)	193(84%)	36(16%)	1.98(1.20-3.26)	0.008	10.20(4.86-21.42)	<0.001
Protocol HR	44(41%)	63(59%)	107	1(8%)	12(92%)	15(34%)	29(66%)	28(56%)	22(44%)	2.50(1.08-5.79)	0.033	15.13(1.82-125.59)	0.012

[a] As in initial genome-wide analysis, performed for induction/consolidation (protocols IA/IB), individuals of the other protocol elements with bilirubin toxicity(CTC grades 1-4) were considered as cases and compared to control patients with normal bilirubin levels(CTC grade 0). Immunophenotype information was not available for 5 of 650 individuals in the discovery cohort and they were therefore excluded from the analysis. Toxicity grading was according to the NCI Common Toxicity Criteria (CTC, version 2).

[b] The amount of controls (CTR) and cases (CAS) per specified rs6744284 genotype is given as indicated: homozygotes for the risk/minor allele (TT), heterozygotes (TC) and homozygotes for the major allele (CC).

[c] Genotypic association was analyzed using binary logistic regression with adjustment for age and immunophenotype. Odds ratios (OR) are listed for heterozygous (het) and homozygous genotype (hom).

Supplementary Table 9. Genotypic association of rs6744284 with hyperbilirubinemia phenotype stratified for potential effect modifiers.

		Amount of included patients		Genotype counts[a]					
		Controls	Cases	Controls	Cases	OR_{het} (95% CI)[b]	P^c	OR_{hom} (95% CI)[b]	P^c
Age (n=650)	Total	215	435	7/81/127	63/195/177	1.73(1.22-2.44)	0.002	6.46(2.86-14.57)	<0.00
	<6	143	193	5/59/79	26/91/76	1.60(1.02-2.53)	0.042	5.41(1.97-14.8)	0.001
	≥6	72	242	2/22/48	37/104/101	2.25(1.27-3.99)	0.006	8.79(2.03-38)	0.004
Immunophenotype (n=645)[d]	Total	214	431	7/80/127	63/192/176	1.73(1.22-2.45)	0.002	6.49(2.88-14.65)	<0.00
	B cell ALL	182	330	6/72/104	50/143/137	1.51(1.03-2.21)	0.035	6.33(2.61-15.32)	<0.00
	T cell ALL	32	101	1/8/23	13/49/39	3.61(1.46-8.95)	0.006	7.67(0.94-62.5)	0.057

[a] Counts of the risk and minor allele homozygotes (TT), heterozygotes (TC) and of the major allele homozygotes (CC).

[b] Odds ratios (OR) for the heterozygous (het) and homozygous(hom) genotype.

[c] *P*-values resulting from logistic regression analysis.

[d] Immunophenotype information was only available for 645 patients of the discovery cohort.

Supplementary Table 10. Characteristics of acute lymphoblastic leukemia (ALL) patients included in the replication cohort (n=224).

		**Patients without hyperbilirubinemia (n=79) n(%)**	**Patients with hyperbilirubinemia (n=145) n(%)**	***P*[a]**
Sex	Male	41 (52%)	88 (61%)	
	Female	38 (48%)	57 (39%)	0.203
Age at diagnosis of ALL [y]	<6	63 (80%)	93 (64%)	
	≥6 <10	12 (15%)	31 (21%)	
	≥10	4 (5%)	21 (14%)	0.032
Immunophenotype	B cell ALL	77 (97%)	139 (96%)	-
	Other/not characterized[b]	2 (3%)	6 (4%)	
White blood cell count at diagnosis of ALL [/µL]	<10000	46 (58%)	82 (57%)	
	≥10000 <50000	22 (28%)	42 (29%)	
	≥50000 <100000	8 (10%)	13 (9%)	
	≥100000	3 (4%)	8 (6%)	0.934
CNS positivity[c]	No	77 (97%)	135 (93%)	
	Yes	0 (0%)	3 (2%)	0.193
	Unknown	2 (3%)	7 (5%)	
Hyperdiploidy[d]	No	57 (72%)	100 (69%)	
	Yes	2 (3%)	7 (5%)	0.391
	Unknown	20 (25%)	38 (26%)	
Prednisone response[e]	Good	78 (99%)	143 (99%)	
	Poor	1 (1%)	2 (1%)	0.944
MRD risk group[f]	Standard	50 (63%)	95 (66%)	
	Intermediate	27 (34%)	43 (30%)	
	High	0 (0%)	1 (1%)	0.637
	Unknown	2 (3%)	6 (4%)	
Final risk group[g]	Standard	42 (53%)	72 (50%)	
	Intermediate	36 (46%)	70 (48%)	
	High	1 (1%)	3 (2%)	0.824
Maximum transaminase levels during protocols IA/IB[h]	CTC grade 0	7 (9%)	3 (2%)	
	CTC grades 1-2	41 (52%)	70 (48%)	
	CTC grades 3-4	31 (39%)	72 (50%)	0.036

Abbreviations: CNS: central nervous system; CTC: Common Toxicity Criteria of the National Cancer Institute version 2.

[a] *P*-values resulting from X^2 or Fisher's exact test: Patients with hyperbilirubinemia, i.e. bilirubin levels >17.1 µmol/L during induction/consolidation (protocols IA/IB) of the ALL therapy (CTC grades 1-4) versus patients with normal levels ≤17.1 µmol/L (CTC grade 0). This replication cohort was genotyped on Affymetrix Genome-wide Human SNP Arrays 5.0 (Affymetrix, South San Francisco, CA, USA) as previously described(10).

[b] No immunophenotype information was available for eight patients.

[c] CNS negative, puncture nontraumatic without leukemic blasts in the cerebrospinal fluid (CSF) after cytocentrifugation; CNS positive, puncture nontraumatic with >5 leukocytes/µL in the CSF with identifiable blasts.

[d] Defined by cytogenetics (>50 chromosomes) or by flow cytometric analyses of the ratio of DNA content of leukemic G0/G1 cells to normal diploid lymphocytes (≥1.16).

[e] Good <1000 leukemic blasts/µL peripheral blood on treatment day 8; poor ≥1000 blasts/µL.

[f] Risk stratification based on minimal residual disease (MRD) analysis for ERG: Standard risk, MRD-negative on treatment day33 and 78; high risk, leukemic cell load ≥$5x10^{-4}$ on treatment day 78; all other results correspond to intermediate risk.

[g] Treatment group based according to risk stratification including all relevant diagnostic parameters.

[h] Toxicity grading of the alanine and aspartate transaminase(ALT/AST) serum levels, during induction/consolidation (protocols IA/IB), was according to CTC, considering 20 U/L as the UNL.

Supplementary Table 11. Characteristics of acute lymphoblastic leukemia (ALL) patients included in subsequent U*GT1A1**28/*37 genotyping (n=544).

		**Patients without hyperbilirubinemia** (n=184)	n(%)	**Patients with hyperbilirubinemia** (n=360)	n(%)	***P***[a]
Sex	Male	101	(55%)	214	(59%)	
	Female	83	(45%)	146	(41%)	0.309
Age at diagnosis of ALL [y]	<6	121	(66%)	159	(44%)	
	≥6 <10	33	(18%)	70	(19%)	
	≥10	30	(16%)	131	(36%)	<0.001
Immunophenotype	B cell ALL	154	(84%)	269	(75%)	
	T cell ALL	30	(16%)	89	(25%)	0.023
	Other/not characterized[b]	0	(0%)	2	(1%)	
White blood cell count at diagnosis of ALL [/µL]	<10000	72	(39%)	149	(41%)	
	≥10000 <50000	69	(38%)	118	(33%)	
	≥50000 <100000	21	(11%)	38	(11%)	
	≥100000	22	(12%)	55	(15%)	0.578
CNS positivity[c]	No	175	(95%)	325	(90%)	
	Yes	6	(3%)	16	(4%)	0.456
	Unknown	3	(2%)	19	(5%)	
Hyperdiploidy[d]	No	106	(58%)	213	(59%)	
	Yes	36	(20%)	55	(15%)	0.263
	Unknown	42	(23%)	92	(26%)	
ETV6-RUNX1 rearrangement	Negative	165	(90%)	315	(88%)	
	Positive	2	(1%)	6	(2%)	0.579
	Unknown	17	(9%)	39	(11%)	
Prednisone response[e]	Good	164	(89%)	305	(85%)	
	Poor	20	(11%)	51	(14%)	0.260
	Unknown	0	(0%)	4	(1%)	
MRD risk group[f]	Standard	67	(36%)	144	(40%)	
	Intermediate	95	(52%)	165	(46%)	
	High	17	(9%)	37	(10%)	0.504
	Unknown	5	(3%)	14	(4%)	
Final risk group[g]	Standard	46	(25%)	104	(29%)	
	Intermediate	103	(56%)	184	(51%)	
	High	35	(19%)	72	(20%)	0.529
Initial bilirubin levels [µmol/L][h]	≤17.1	101	(55%)	202	(56%)	
	>17.1	2	(1%)	18	(5%)	0.030
	Unknown	81	(44%)	140	(39%)	
Maximum transaminase levels in protocols IA/IB[i]	CTC grade 0	25	(14%)	14	(4%)	
	CTC grades 1-2	85	(46%)	159	(44%)	
	CTC grades 3-4	72	(39%)	187	(52%)	<0.001
	Unknown	2	(1%)	0	(0%)	
*UGT1A1**28/*37 genotype[j]	(*1/*1)	98	(53%)	133	(37%)	
	(*1/*28) or (*1/*37)	79	(43%)	172	(48%)	
	(*28/*28) or (*37/*37)	7	(4%)	55	(15%)	<0.001
rs6744284 genotype[k]	CC	111	(60%)	152	(42%)	
	TC	69	(38%)	159	(44%)	
	TT	4	(2%)	49	(14%)	<0.001

Abbreviations: ALL: acute lymphoblastic leukemia; CNS: central nervous system; CTC: Common Toxicity Criteria of the National Cancer Institute version 2 GWAS: genome-wide association study.

[a] *P*-values resulting from X^2 or Fisher's exact test comparing the GWAS phenotype groups: patients with normal bilirubin levels(CTC grade 0, controls) versus patients with elevated bilirubin levels (CTC grades 1-4, cases) during induction/consolidation (protocols IA/IB). Toxicity grading was according to CTC.

[b] One patient was diagnosed with acute undifferentiated leukemia and no immunophenotype information was available for a second patient.

[c] CNS negative, puncture nontraumatic without leukemic blasts in the cerebrospinal fluid (CSF) after cytocentrifugation; CNS positive, puncture nontraumatic with >5 leukocytes/µL in the CSF with identifiable blasts.

[d] Defined by cytogenetics (>50 chromosomes) or by flow cytometric analyses of the ratio of DNA content of leukemic G0/G1 cells to normal diploid lymphocytes (≥1.16).

[e] Good <1000 leukemic blasts/µL peripheral blood on treatment day 8; poor ≥1000 blasts/µL.

[f] Risk stratification based on minimal residual disease (MRD) analysis for ERG: Standard risk, MRD-negative on treatment day33 and 78; high risk, leukemic cell load $\geq 5 \times 10^{-4}$ on treatment day 78; all other results correspond to intermediate risk.

[g] Treatment group based according to risk stratification including all relevant diagnostic parameters.

[h] Total serum bilirubin levels at day 0 of the therapy; levels ≤17.1 µmol/L corresponding to the upper normal level (UNL) and CTC grade 0.

[i] Toxicity grading of the alanine and aspartate transaminase (ALT/AST) serum levels was according to CTC, considering 20 U/L as the UNL.

[j] In rs3064744 genotyping we analyzed *UGT1A1**28 and *37, with 7 (*UGT1A1**28) and 8 (*37) instead of 6 (*1) thymine-adenine repeats (9-12). The here applied assay could not distinguish between *28 and *37, but *37 is almost absent in European populations(10). The amount of controls and cases per specified genotype is given as indicated: homozygotes for the risk/minor allele (*28/*28) or (*37/*37), heterozygotes (*1/*28) or (*1/*37) and homozygotes for the major allele (*1/*1).

[k] The amount of controls and cases per specified rs6744284 genotype is given as indicated: homozygotes for the risk/minor allele (TT), heterozygotes (TC) and homozygotes for the major allele (CC).

Supplementary Table 12. Association of identified risk loci and known Gilbert's syndrome related variants with hyperbilirubinemia.

Variant Identifier	Risk allele (A1)[a]	Risk allele frequency[b]		Allelic association[c]			Frequency of CTR/CAS per A1A1(%)[d]		Frequency of CTR/CAS per A1A2(%)[d]		Frequency of CTR/CAS per A2A2(%)[d]		Genotypic association[e]			
		Cases	Controls	X^2	OR(95% CI)	$P(X^2)$	CTR	CAS	CTR	CAS	CTR	CAS	OR_{het}(95% CI)	P	OR_{hom}(95% CI)	P
rs6744284	T	0.369	0.221	29.0	2.06(1.6-2.7)	$7.3x10^{-8}$	7(10%)	63(90%)	81(29%)	195(71%)	127(42%)	177(58%)	1.73(1.22-2.44)	0.002	6.46(2.86-14.57)	<0.001
rs6715829	T	0.414	0.260	29.3	2.00(1.6-2.6)	$6.3x10^{-8}$	13(15%)	75(85%)	86(29%)	210(71%)	116(44%)	150(56%)	1.89(1.33-2.68)	<0.001	4.46(2.36-8.43)	<0.001
rs17868323	G	0.658	0.574	8.5	1.42(1.1-1.8)	$3.5x10^{-3}$	70(27%)	188(73%)	107(35%)	196(65%)	38(43%)	51(57%)	1.36(0.84-2.21)	0.206	2.00(1.21-3.3)	0.007
rs3806596	C	0.502	0.414	9.0	1.43(1.1-1.8)	$2.7x10^{-3}$	33(23%)	110(77%)	112(34%)	217(66%)	70(39%)	108(61%)	1.26(0.86-1.83)	0.237	2.16(1.32-3.53)	0.002
rs3064744[f]	*28/*37	0.392	0.253	20.8	1.90(1.4-2.5)	$5.0x10^{-6}$	7(11%)	55(89%)	79(31%)	172(69%)	98(42%)	133(58%)	1.60(1.1-2.33)	0.013	5.79(2.53-13.26)	<0.001

[a] Risk allele, was equal to minor allele for rs6744284, rs6715829, rs3806596 and rs3064744 (*UGT1A**28/*37). For rs17868323 the risk mediating allele was the major and alternate allele 'G' for the European population.

[b] Risk allele frequency as detected for cases and controls.

[c] Allelic association calculated without covariate adjustment. Information was available for 650 patients (435 cases, 215 controls) for all genotyped and imputed variants and for 544 patients (360 cases, 184 controls) typed for the *UGT1A**28/*37 variation.

[d] The amount of controls (CTR) and cases (CAS) per specified rs6744284 genotype is given as indicated: homozygotes for the risk/minor allele (A1A1), heterozygotes (A1A2) and homozygotes for the major allele (A2A2).

[e] Genotypic association calculated using binary logistic regression without covariate adjustment. Information was available for 650 patients for the genotyped and imputed variants (GWAS SNV) and for 544 patients typed separately for the *UGT1A**28/*37 variation. Resulting odds ratios (OR) are given for homozygous (OR_{hom}) and heterozygous genotype (OR_{het}).

[f] In rs3064744 genotyping we analyzed *UGT1A1**28 and *37, with 7 (*UGT1A1**28) and 8 (*37) instead of 6 (*1) thymine-adenine repeats(11-14). The here applied assay could not distinguish between *28 and *37, but *37 is almost absent in European populations(12).

Supplementary Table 13. Correlation of rs6744284 with imputed top SNV and *UGT1A* variations related to hyperbilirubinemia and the Gilbert's syndrome.

		Variant Identifier	Risk allele	Consequence	Spearman's correlation[a] ρ	r^2	P	Amount of tested patients	Included rs6744284 (TT)	Homozygotes for both variants
**Top SNV imputed**		rs6715829	T	intronic	0.828	0.686	$8.7x10^{-165}$	650	70	64 (91%)
***UGT1A* haplotype[b]**		rs3806596	C	*UGT1A3* -66T>C	0.647	0.419	$2.9x10^{-78}$	650	70	65 (93%)
	***UGT1A6*2a*[c]**	rs6759892	G	p.S7A	0.711	0.506	$4.2x10^{-100}$	644	70	66 (94%)
		rs2070959	G	p.T181A	0.812	0.659	$1.3x10^{-153}$	650	70	63 (90%)
		rs1105879	C	p.R184S	0.800	0.640	$1.3x10^{-144}$	644	70	66 (94%)
	***UGT1A7*3*[d]**	rs17868323	G	p.N129K	0.434	0.188	$3.5x10^{-31}$	650	70	68 (97%)
		rs7586110	G	*UGT1A7* -57T>G	0.714	0.510	$2.4x10^{-102}$	650	70	63 (90%)
		rs11692021	C	p.W208R	0.714	0.510	$2.4x10^{-102}$	650	70	63 (90%)
	***UGT1A1**28/*37**	rs3064744	TA/TATA	dupTA/dupTATA	0.836	0.699	$1.7x10^{-143}$	544	53	49 (92%)

[a] Genotype correlation was assessed according to Spearman; resulting correlation coefficient (ρ) and coefficient of determination (r^2) are indicated.

[b] Correlation of *UGT1A1* haplotype to rs6744284 TT genotype was: ρ= 0.782, r^2= 0.612 and P=$3.5x10^{-112}$. Patients positive for this haplotype carry homozygously the risk alleles of *UGT1A1**28/*37, *UGT1A6*2a* and *UGT1A7*3* and rs3806596. Only patients with complete information were included (n= 538, 54 *UGT1A* homozygotes, 53 rs6744284 TT homozygotes and 43 individuals homozygous for both).

[c] Correlation of *UGT1A6*2a* haplotype to rs6744284 TT genotype was: ρ= 0.798, r^2= 0.637 and P=$2.6x10^{-143}$(n= 644, 84 *UGT1A6*2a* homozygotes, 70 rs6744284 TT homozygotes and 63 individuals homozygous for both).

[d] Correlation of *UGT1A7*3* haplotype to rs6744284 TT genotype was: ρ= 0.677, r^2= 0.677 and P=$2.3x10^{-88}$(n= 650, 110 *UGT1A7*3* homozygotes, 70 rs6744284 TT homozygotes and 63 individuals homozygous for both).

Supplementary information on the correlation analysis of known Gilbert's syndrome (GS) related variations with rs6744284

We assessed the correlation of rs6744284 with *UGT1A1*28*/*37 and further eight GS-related variations (13, 15, 16), to proof for extended haplotypes: *UGT1A7*3 (rs17868323, rs7586110 and rs11692021), UGT1A6*2a (rs6759892, rs2070959 and rs1105879) and UGT1A1 (UGT1A6*2a, UGT1A7*3, rs3806596 and UGT1A1*28)*. The correlations between rs6744284 and the individual variants ranged from moderate (rs17868323, ρ=0.434, r^2=0.188, P=3.5x10^{-31}) to high (*UGT1A1*28*/*37 ρ=0.836, r^2=0.699, P=1.7x10^{-143}); see Suppl. Table 13. Two included variants, rs2070959 (p.T181A, OR=1.9, P=1.0x10^{-5}) and rs1105879 (p.R184S, OR=1.8, P=7.1x10^{-7}) were among the twenty most associated ones of our GWAS and *in silico* prediction indicated a probably damaging effect for rs1105879 ("Sorting Intolerant From Tolerant", SIFT, score=0.1). Nevertheless, none of these SNV showed a stronger association with hyperbilirubinemia than rs6744284.

Supplementary information on the impact of hyperbilirubinemia on therapy delays in the discovery cohort

The median time to protocol day 78 patients of the discovery cohort with available information (n=634) needed was 89±10 days (range 70-154 days). The delays related to the bilirubin levels during this period differed, but not significantly (P=0.072): patients without hyperbilirubinemia needed 90 days (range 72-141 days) to complete consolidation, while patients with moderate and high hyperbilirubinemia needed 89 (range 70-154 days) and 91 days (range 76-146 days), respectively.

Supplementary Table 14. Clinical characteristics of the acute lymphoblastic leukemia (ALL) patients of the GWAS discovery cohort according to the severity of bilirubin toxicity during induction/consolidation (protocols IA/IB, n=650).

		**CTC grade 0** n=215(n%)	**CTC grades 1-2** n=367(n%)	**CTC grades 3-4** n=68(n%)	***P***[a]
Sex	Male	120(56%)	208(57%)	45(66%)	
	Female	95(44%)	159(43%)	23(34%)	0.295
Age at diagnosis of ALL [years]	<6	143(67%)	180(49%)	13(19%)	
	≥6 <10	35(16%)	77(21%)	11(16%)	
	≥10	37(17%)	110(30%)	44(65%)	<0.001
Immunophenotype	B cell ALL	182(85%)	277(75%)	53(78%)	
	T cell ALL	32(15%)	86(23%)	15(22%)	0.041
	Other/not characterized[b]	1(0%)	4(1%)	0(0%)	
White blood cell count at diagnosis of ALL[/µL]	<10000	83(39%)	149(41%)	30(44%)	
	≥10000 <50000	83(39%)	121(33%)	21(31%)	
	≥50000 <100000	24(11%)	44(12%)	6(9%)	
	≥100000	25(12%)	53(14%)	11(16%)	0.730
CNS positivity[c]	No	201(93%)	336(92%)	59(87%)	
	Yes	7(3%)	11(3%)	7(10%)	0.016
	Unknown	7(3%)	20(5%)	2(3%)	
Hyperdiploidy[d]	No	122(57%)	212(58%)	42(62%)	
	Yes	44(20%)	57(16%)	8(12%)	0.222
	Unknown	49(23%)	98(27%)	18(26%)	
ETV6-RUNX1 rearrangement	Negative	191(89%)	309(84%)	61(90%)	
	Positive	4(2%)	18(5%)	0(0%)	0.035
	Unknown	20(9%)	40(11%)	7(10%)	
Prednisone response[e]	Good	194(90%)	313(85%)	60(88%)	
	Poor	21(10%)	50(14%)	6(9%)	0.268
	Unknown	0(0%)	4(1%)	2(3%)	
MRD risk group[f]	Standard	74(34%)	141(38%)	26(38%)	
	Intermediate	117(54%)	180(49%)	33(49%)	
	High	17(8%)	33(9%)	5(7%)	0.788
	Unknown	7(3%)	13(4%)	4(6%)	
Final risk group[g]	Standard	50(23%)	107(29%)	18(26%)	
	Intermediate	127(59%)	189(51%)	39(57%)	
	High	38(18%)	71(19%)	11(16%)	0.447
Initial bilirubin levels [µmol/L][h]	≤17.1	114(53%)	202(55%)	31(46%)	
	>17.1	2(1%)	12(3%)	7(10%)	0.001
	Unknown	99(46%)	153(42%)	30(44%)	
Maximum transaminase levels in protocols IA/IB[i]	CTC grade 0	29(13%)	14(4%)	1(1%)	
	CTC grades 1-2	96(45%)	177(48%)	16(24%)	
	CTC grades 3-4	88(41%)	176(48%)	51(75%)	<0.001
	Unknown	2(1%)	0(0%)	0(0%)	
Maximum bilirubin levels during protocol IA[j]	CTC grade 0	199 (93%)	54 (15%)	1 (1%)	
	CTC grades 1-2	0 (0%)	305 (83%)	8 (12%)	
	CTC grades 3-4	0 (0%)	0 (0%)	59 (87%)	<0.001
	Unknown	16 (7%)	8 (2%)	0 (0%)	
Maximum bilirubin levels during protocol IB[j]	CTC grade 0	199 (93%)	135 (37%)	18 (26%)	
	CTC grades 1-2	0 (0%)	221 (60%)	27 (40%)	
	CTC grades 3-4	0 (0%)	0 (0%)	21 (31%)	<0.001
	Unknown	16 (7%)	11 (3%)	2 (3%)	
Maximum bilirubin levels during entire therapy[k]	CTC grade 0	166 (77%)	0 (0%)	0 (0%)	
	CTC grades 1-2	47 (22%)	346 (94%)	0 (0%)	
	CTC grades 3-4	2 (1%)	21 (6%)	68 (100%)	<0.001

Abbreviations: CNS: central nervous system; CTC: Common Toxicity Criteria of the National Cancer Institute version 2; GWAS: genome-wide association study.

[a] *P*-values resulting from X^2 tests: Patients with normal bilirubin levels(CTC grade 0, ≤17.1 µmol/L(UNL)) during induction/consolidation (protocols IA/IB) of the ALL therapy were compared to patients with moderate bilirubin levels (grades 1-2) and high levels (grades 3-4). Toxicity grading was according to CTC.

[b] One patient was diagnosed with acute undifferentiated leukemia and no immunophenotype information was available for four patients.

[c] CNS negative, puncture nontraumatic without leukemic blasts in the cerebrospinal fluid (CSF) after cytocentrifugation; CNS positive, puncture nontraumatic with >5 leukocytes/µL in the CSF with identifiable blasts.

[d] Defined by cytogenetics (>50 chromosomes) or by flow cytometric analyses of the ratio of DNA content of leukemic G0/G1 cells to normal diploid lymphocytes (≥1.16).

[e] Good <1000 leukemic blasts/µL peripheral blood on treatment day 8; poor ≥1000 blasts/µL.

[f] Risk stratification based on minimal residual disease (MRD) analysis for ERG: Standard risk, MRD-negative on treatment day33 and 78; high risk, leukemic cell load $\geq 10^{-3}$ on treatment day 78; all other results correspond to intermediate risk.

[g] Treatment group based according to risk stratification including all relevant diagnostic parameters.

[h] Total serum bilirubin levels at day 0 of the therapy; levels ≤17.1 µmol/L correspond to the UNL and CTC grade 0.

[i] Toxicity grading of the alanine and aspartate transaminase serum activity levels during induction/consolidation (protocols IA/IB) was according to CTC, considering 20 U/L as the UNL.

[j] Bilirubin toxicity grading during induction/consolidation (protocols IA/IB) was according to the CTC, with grade 0 corresponding to total serum levels ≤UNL, grade 1 to levels >UNL to 1.5xUNL, grade 2 levels >1.5x UNL to 3.0x UNL, grade 3 levels >3.0x UNL to 10.0x UNL and grade 4 to levels >10.0x UNL.

[k] The highest individual bilirubin toxicity level throughout the entire treatment course under investigation (Suppl. Figure 3). Toxicity grading was as above (CTC).

Supplementary Table 15. Estimated hazard ratios from the multivariable Cox proportional model on the hazard of relapse in patients with high hyperbilirubinemia (≥CTC grade 3) during induction and/or consolidation (n=68).

Variable	Hazard Ratio (95% CI)[a]	*P*(X^2)
rs6744284 TT[b]	0.07 (0.01-0.65)	0.020
ALT/AST CTC grade 4[c]	0.65 (0.14-3.01)	0.578
MRD standard risk[d]	1.00 (0.29-3.47)	0.999
MRD high risk[d]	2.48 (0.16-38.46)	0.516
Slow early response[e]	0.15 (0.02-1.50)	0.106
Initial WBC count ≥100000[f]	13.00 (3.03-55.68)	0.001

[a] Hazard ratios (HR) are given as indicated with the corresponding 95% confidence intervals (95% CI).

[b] HR compared the presence of rs6744284 TT genotype with wild type (CC) or heterozygous(TC) genotype.

[c] HR compared patients with severe alanine (ALT) or aspartate (AST) transaminase serum levels ≥CTC grade 4 with patients presenting normal or moderately elevated levels.

[d] Minimal residual disease (MRD) standard risk, negative on treatment days 33 and 78; MRD high risk, leukemic cell load ≥$5x10^{-4}$ on treatment day 78; all other results MRD intermediate risk. HR compared with the other respective MRD groups.

[e] MRD ≥$5x10^{-4}$ on treatment day 33 and positivity of <$5x10^{-4}$ on treatment day 78. HR compared with MRD intermediate-risk patients with no slow early response.

[f] HR compared patients with a white blood cell (WBC) counts at diagnosis ≥100,000 /µL with patients presenting WBC counts <100,000 /µL.

Supplementary References

1. National Cancer Institute. Common toxicity criteria version 2 1998 [Available from: https://ctep.cancer.gov/protocolDevelopment/electronic_applications/ctc.htm.

2. Browning SR, Browning BL. Rapid and accurate haplotype phasing and missing-data inference for whole-genome association studies by use of localized haplotype clustering. Am J Hum Genet. 2007;81(5):1084-97.

3. Diabetes Genetics Initiative of Broad Institute of H, Mit LU, Novartis Institutes of BioMedical R, Saxena R, Voight BF, Lyssenko V, et al. Genome-wide association analysis identifies loci for type 2 diabetes and triglyceride levels. Science. 2007;316(5829):1331-6.

4. Kruskal WH, Wallis WA. Use of Ranks in One-Criterion Variance Analysis. Journal of the American Statistical Association. 1952;47(260):583-621.

5. Kaplan EL, Meier P. Nonparametric Estimation from Incomplete Observations. Journal of the American Statistical Association. 1958;53(282):457-81.

6. Kalbfleish P. Statistical analysis of failure time data. Statistical analysis of failure time data. 1st ed. New York: John Wiley& Sons; 1980. p. 163.

7. Gray RJ. A Class of K-Sample Tests for Comparing the Cumulative Incidence of a Competing Risk. The Annals of Statistics. 1988;16(3):1141-54.

8. Cox DR. Regression Models and Life-Tables. Journal of the Royal Statistical Society Series B (Methodological). 1972;34(2):187-220.

9. Migliorini G, Fiege B, Hosking FJ, Ma Y, Kumar R, Sherborne AL, et al. Variation at 10p12.2 and 10p14 influences risk of childhood B-cell acute lymphoblastic leukemia and phenotype. Blood. 2013;122(19):3298-307.

10. Ellinghaus E, Stanulla M, Richter G, Ellinghaus D, te Kronnie G, Cario G, et al. Identification of germline susceptibility loci in ETV6-RUNX1-rearranged childhood acute lymphoblastic leukemia. Leukemia. 2012;26(5):902-9.

11. Bosma PJ, Chowdhury JR, Bakker C, Gantla S, de Boer A, Oostra BA, et al. The genetic basis of the reduced expression of bilirubin UDP-glucuronosyltransferase 1 in Gilbert's syndrome. N Engl J Med. 1995;333(18):1171-5.

12. Beutler E, Gelbart T, Demina A. Racial variability in the UDP-glucuronosyltransferase 1 (UGT1A1) promoter: a balanced polymorphism for regulation of bilirubin metabolism? Proc Natl Acad Sci U S A. 1998;95(14):8170-4.

13. Lampe JW, Bigler J, Horner NK, Potter JD. UDP-glucuronosyltransferase (UGT1A1*28 and UGT1A6*2) polymorphisms in Caucasians and Asians: relationships to serum bilirubin concentrations. Pharmacogenetics. 1999;9(3):341-9.

14. Strassburg CP. Pharmacogenetics of Gilbert's syndrome. Pharmacogenomics. 2008;9(6):703-15.

15. Guillemette C, Ritter JK, Auyeung DJ, Kessler FK, Housman DE. Structural heterogeneity at the UDP-glucuronosyltransferase 1 locus: functional consequences of three novel missense mutations in the human UGT1A7 gene. Pharmacogenetics. 2000;10(7):629-44.

16. Ehmer U, Kalthoff S, Fakundiny B, Pabst B, Freiberg N, Naumann R, et al. Gilbert syndrome redefined: a complex genetic haplotype influences the regulation of glucuronidation. Hepatology. 2012;55(6):1912-21.

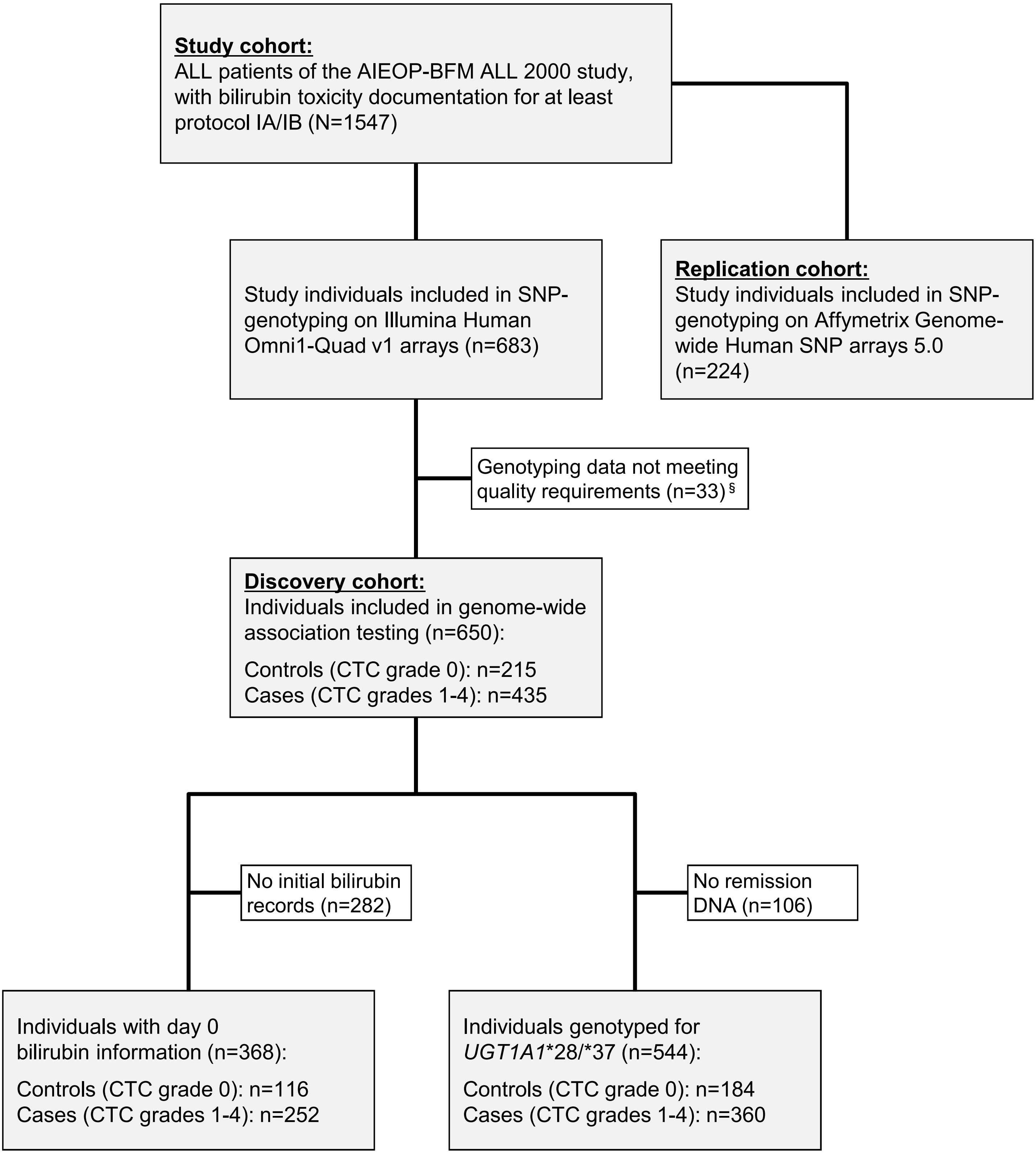

Supplementary Figure 1. Consolidated Standards of Reporting Trials (CONSORT) diagram of inclusion criteria for the study population. Bilirubin toxicity grading was according to the Common Toxicity criteria (CTC) of the National Cancer Institute, version 2, considering 17.1 µmol/L as the upper normal limit (UNL). § We excluded a total of 33 patients: 24 patients with a poor genotype call rate (<98%)/outlying heterozygosity rate, 3 patients with divergent sex information, 1 patient for cryptical familiar relationship (Proportion IBD>0.2) and 6 patients for non-European ancestry; one patient met two criteria (outlying heterozygosity rate and cryptic familiar relationship).

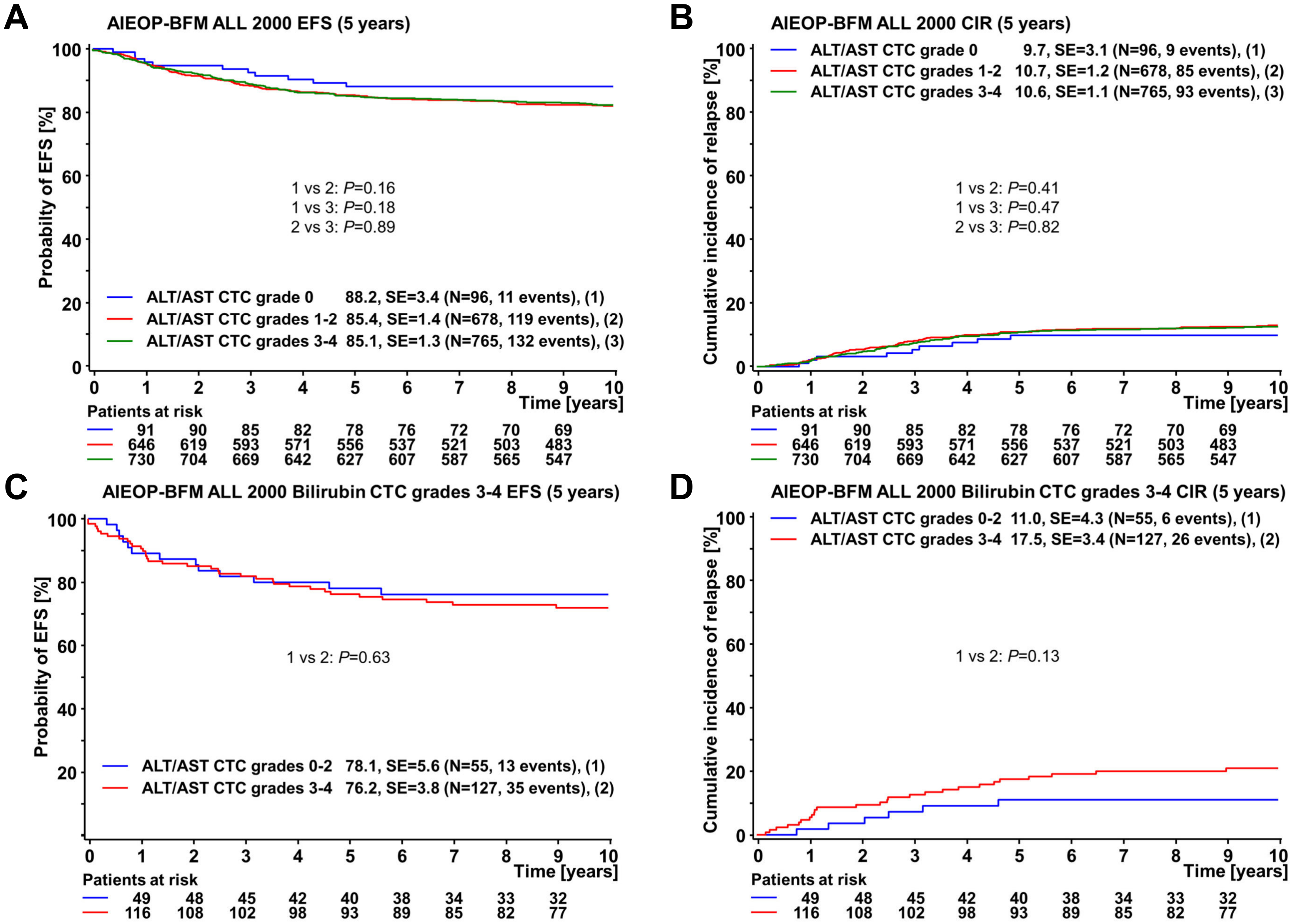

Supplementary Figure 2. Estimated 5-year event-free survival (EFS) and cumulative incidence of relapse (CIR) at 5 years in the study cohort by the maximum transaminase levels during induction/consolidation (protocols IA/IB) [%]. For panels (A) and (B) the maximum alanine (ALT) and/or aspartate (AST) transaminase levels were included. Plots (C) and (D) show the effect of concurrent high bilirubin and transaminase levels, ≥ CTC grade 3, during protocols IA/IB compared with lower or normal levels. Toxicity gradings are given according to the Common Toxicity Criteria (CTC) of the National Cancer Institute, version 2; standard error (SE) and the amount of included individuals (N) are indicated for each category.

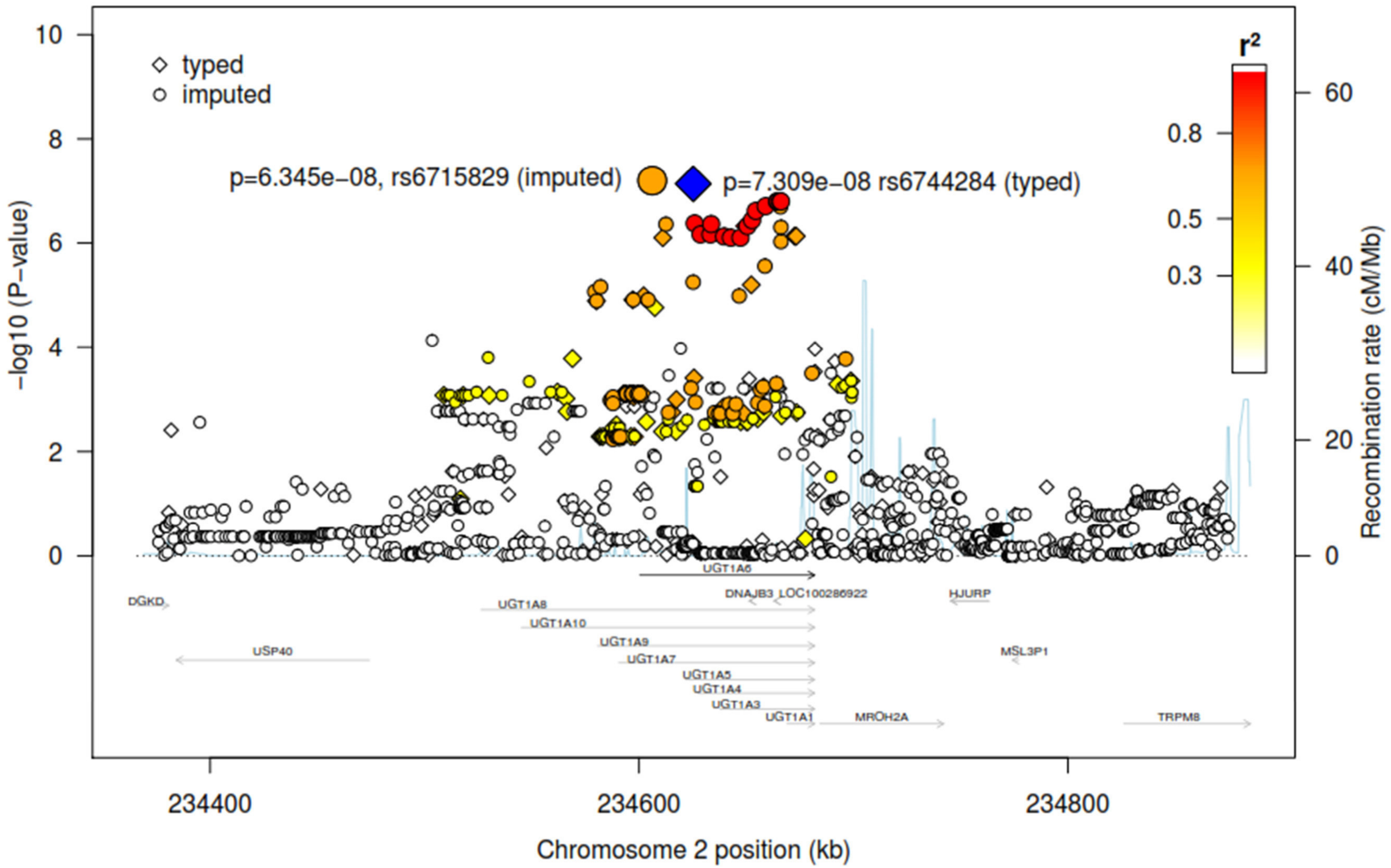

Supplementary Figure 3. Regional plot of association results and recombination rates for the identified risk locus in the *UGT1A* region (2q37). The plot shows the allelic association as −log10 *P*-values (left y-axis) of genotyped (rhombs) and imputed (circles) SNV in the GWAS samples and the recombination rates (right y-axis). Plotting was restricted to a window of ±500kb around the index SNV, rs6744284. The allelic association *P*-values of the typed (large blue rhomb) and the imputed lead SNV (large orange circle) are indicated. The magnitude of linkage disequilibrium (LD) with the typed lead SNV measured by r^2 is reflected by the color of each SNV symbol; for color coding, see upper right corner of the plot. Recombination activity in centimorgans (cM) per megabase (Mb) is depicted by a blue line. Genome coordinates are from NCBI human genome GRCh37. Both top associated variants (typed and imputed) reside within the intronic regions of the overlapping isoforms *UGT1A10*, *9*, *8*, *7* and *6*. Moreover, rs6744284 is also located within the first intron of *UGT1A5* and in the promoter region of *UGT1A4, -*2127 bp upstream to its first exon.

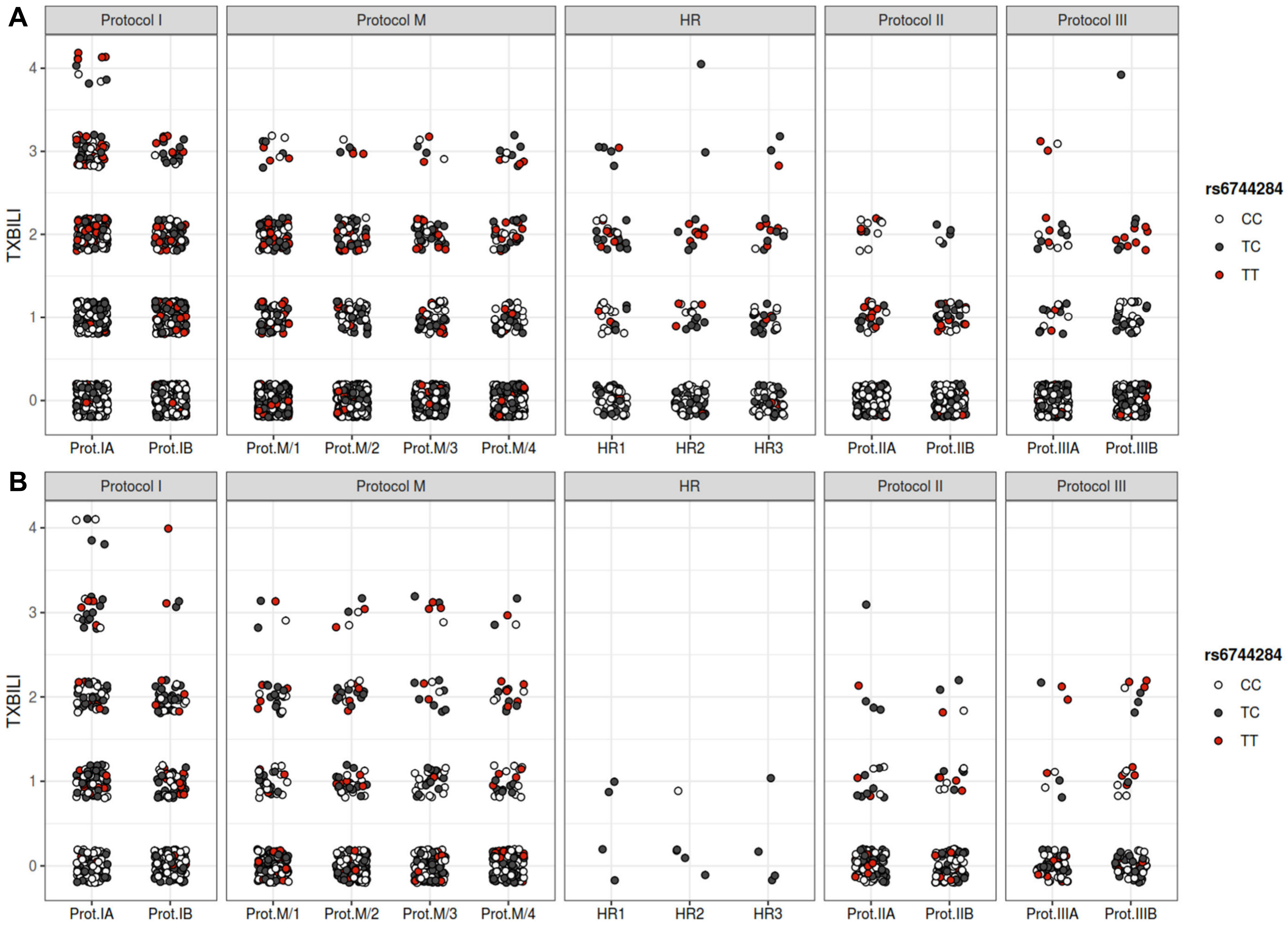

Supplementary Figure 4. Total serum bilirubin levels by treatment element and rs6744284 genotype. Panel (A) shows 4227 bilirubin toxicity (TXBILI) records of the 650 ALL patients included in the discovery cohort; panel (B) 1558 records of the 224 patients of the replication cohort. The rs6744284 genotypes are indicated as follows: red dots represent TT (minor allele), gray dots TC and white dots CC. Toxicity grading was according to the Common Toxicity Criteria (CTC) of the National Cancer Institute, version 2.

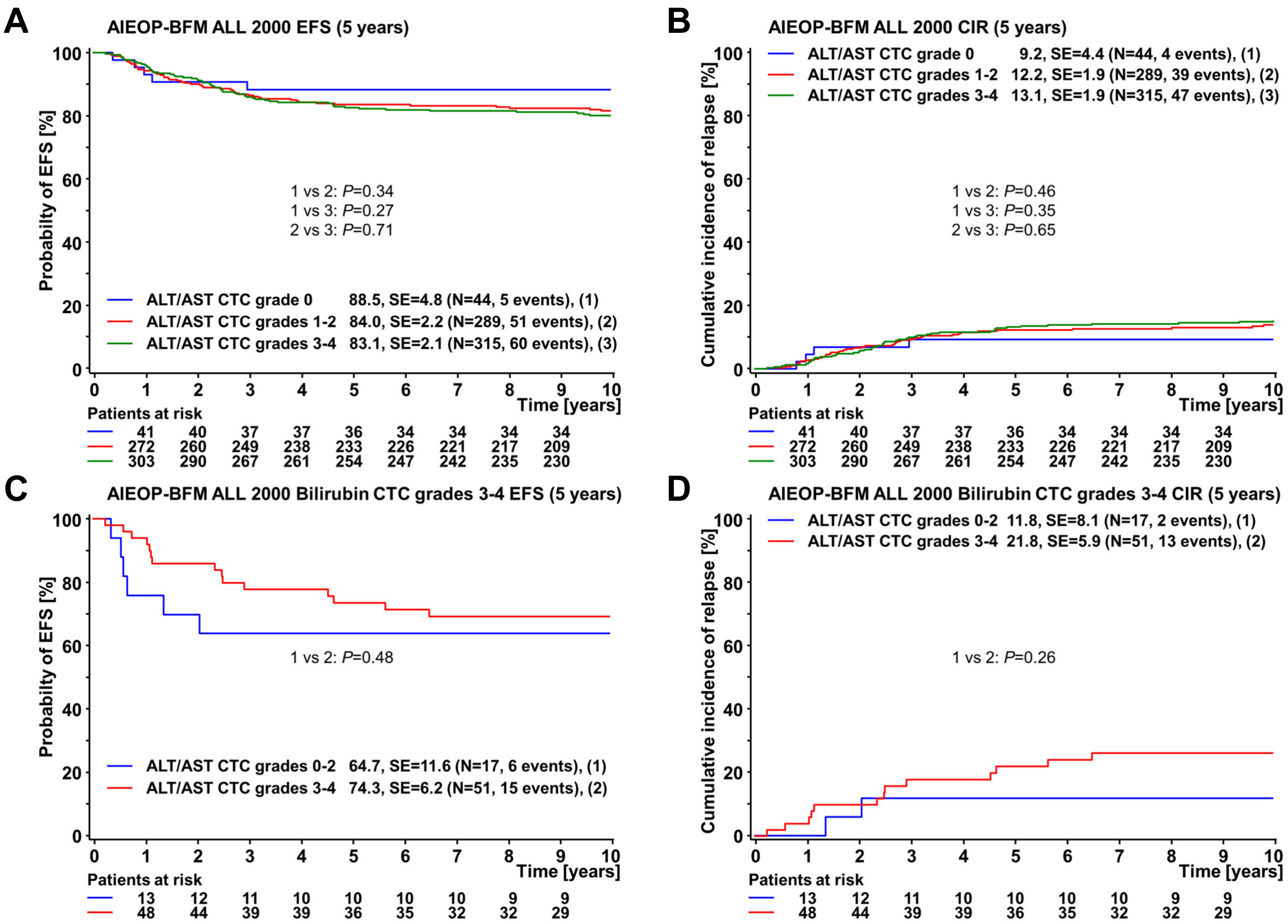

Supplementary Figure 5. Estimated 5-year event-free survival (EFS) and cumulative incidence of relapse (CIR) at 5 years in the discovery cohort by the maximum transaminase levels during induction/consolidation (protocols IA/IB) [%]. For panels (A) and (B) the maximum alanine (ALT) and/or aspartate (AST) transaminase levels were included. Plots (C) and (D) show the effect of concurrent high bilirubin and transaminase levels, ≥ CTC grade 3, during protocols IA/IB compared with lower or normal levels. Toxicity gradings are given according to the Common Toxicity Criteria (CTC) of the National Cancer Institute, version 2; standard error (SE) and the amount of included individuals (N) are indicated for each category.
